# Naturally arising memory-phenotype CD4^+^ T lymphocytes contain an undifferentiated population that can generate T_H_1, T_H_17, and T_reg_ cells

**DOI:** 10.1126/sciadv.adq6618

**Published:** 2024-12-04

**Authors:** Akihisa Kawajiri, Jing Li, Keita Koinuma, Ziying Yang, Hye Jin Yoon, Jaeu Yi, Hiroyuki Nagashima, Minami Ishii, Feng Gao, Kosuke Sato, Shunichi Tayama, Hideo Harigae, Yoichiro Iwakura, Naoto Ishii, Alan Sher, Kazuyoshi Ishigaki, Jinfang Zhu, Kwang Soon Kim, Takeshi Kawabe

**Affiliations:** ^1^ Department of Microbiology and Immunology, Tohoku University Graduate School of Medicine, Sendai, Miyagi, Japan.; ^2^ Department of Hematology, Tohoku University Graduate School of Medicine, Sendai, Miyagi, Japan.; ^3^ Department of Life Sciences, Pohang University of Science and Technology, Pohang, Republic of Korea.; ^4^ Department of Internal Medicine, Division of Rheumatology, Washington University School of Medicine, St. Louis, MO, USA.; ^5^ Department of Biological Science, Ajou University, Suwon, Republic of Korea.; ^6^ Center for Animal Disease Models, Research Institute for Biomedical Sciences, Tokyo University of Science, Noda, Chiba, Japan.; ^7^ Laboratory of Parasitic Diseases, National Institute of Allergy and Infectious Diseases, National Institutes of Health, Bethesda, MD, USA.; ^8^ Laboratory for Human Immunogenetics, RIKEN Center for Integrative Medical Sciences, Yokohama, Kanagawa, Japan.; ^9^ Molecular and Cellular Immunoregulation Section, Laboratory of Immune System Biology, National Institute of Allergy and Infectious Diseases, National Institutes of Health, Bethesda, MD, USA.

## Abstract

Memory-phenotype (MP) CD4^+^ T lymphocytes develop from naïve cells via self-recognition at homeostasis. While previous studies defined MP cells as a heterogeneous population that comprises T helper 1 (T_H_1)/17–like subsets, functional significance of the T-bet^−^ Rorγt^−^ subpopulation remains unknown. Here we show that MP lymphocytes as a whole population can differentiate into T_H_1/17/regulatory T (T_reg_) cells to mediate mild and persistent inflammation in lymphopenic environments, whereas naïve cells exhibit strong, T_H_1-dominated responses. Moreover, we demonstrate that MP lymphocytes comprise not only T_H_1/17-differentiated subsets but a polyclonal, transcriptomically immature “undifferentiated” subpopulation at homeostasis. Furthermore, our data argue that while the T-bet^+^ Rorγt^−^ MP subset is terminally T_H_1-differentiated, its undifferentiated counterpart retains the capacity to rapidly proliferate to differentiate into T_H_1/17/T_reg_ cells, with the latter response tonically constrained by preexisting T_reg_ cells. Together, our results identify undifferentiated MP CD4^+^ T lymphocytes as a unique precursor that has a diverse differentiation potential to generate T_H_1/17/T_reg_ cells to contribute to pathogenesis of inflammation.

## Teaser

Undifferentiated memory-phenotype CD4^+^ T lymphocytes can give rise to T_H_1/17/T_reg_ cells to contribute to inflammatory responses.

## INTRODUCTION

CD4^+^ T lymphocytes are essential for adaptive immune responses. In pathogen infection, naïve CD4^+^ T cells that have T cell receptors (TCRs) specific for foreign antigens presented on major histocompatibility complex class II (MHC II) are activated to differentiate into effector cells. Upon pathogen clearance, most of these cells die, leaving a small fraction of memory cells that can quickly and strongly respond to reinfection with the same pathogen. These CD4^+^ T lymphocyte compartments are strictly regulated homeostatically throughout animal’s life (*1*, *2*).

In unimmunized mice housed in a specific pathogen–free (SPF) environment where immune responses toward explicit foreign antigens are absent, ~10% of Foxp3^−^ CD4^+^ αβT cells in secondary lymphoid tissues have a memory phenotype (i.e., CD44^hi^ CD62L^lo^) (*1*, *2*). This population was previously assumed to represent “authentic” memory cells that are specific for foreign antigens derived from commensal microbiota and/or food (*2*, *3*). However, we and other groups recently reported that the same lymphocyte population equally exists in germ-free (GF) and antigen-free mice that lack both commensal and food antigens (*4*, *5*) and that these CD44^hi^ CD62L^lo^ cells can spontaneously develop from naïve precursors via self-antigen recognition in steady state and exert innate effector function in host defense (*6*). Such naturally arising “memory-phenotype” (MP) CD4^+^ T lymphocytes are now thought to represent a self-antigen–driven T cell subpopulation that can provide lymphocyte-mediated innate immunity together with natural killer (NK), NKT, virtual memory CD8^+^ T, and innate lymphoid cells (ILCs) (*7*–*10*).

Accumulating evidence suggests MP CD4^+^ T lymphocytes as a heterogenous population that contains rapidly proliferating (*11*, *12*), T helper 1 (T_H_1)–differentiated (*6*, *13*), and T_H_17-differentiated (*14*) subsets. In steady state, MP cells comprise major T-bet^+^ and minor Rorγt^+^ subpopulations (*13*, *14*). In their development, MP cells newly generated from naïve precursors extensively proliferate in response to self-antigens as well as costimulatory molecules (*5*, *6*, *15*). With time, these cells gradually cease to divide while acquiring high T-bet expression (*5*, *6*), with the latter response being promoted by interleukin-12 (IL-12) tonically produced by type 1 dendritic cells at low levels (*13*). The resultant T-bet^hi^ subset plays a dominant role in MP cell–mediated innate type 1 responses by producing interferon-γ (IFN-γ) in response to IL-12p70 in the absence of antigen recognition. Similarly, CCR6^hi^ MP cells can exert innate T_H_17-like effector function by secreting IL-17A and granulocyte-macrophage colony-stimulating factor in response to IL-23 and IL-1β (*14*, *16*). However, functional significance of T-bet^−^ Rorγt^−^ MP cells that occupy ~50% of the total MP population but lack innate T_H_1-like and T_H_17-like functions has yet to be determined.

Given their self-reactive as well as proliferative nature, we hypothesized that T-bet^−^ Rorγt^−^ MP cells can contribute to pathogenesis of autoimmune or inflammatory diseases. It is well established that such inflammatory disorders can be induced and/or exaggerated by T lymphocytes in lymphopenic environments both in mice (*17*–*19*) and humans (*20*–*22*). In the mouse, self-antigen–specific TCR-transgenic CD4^+^ and CD8^+^ T cells can induce inflammatory responses under lymphopenic conditions (*19*, *23*, *24*). Similarly, polyclonal naïve (CD45RB^hi^, CD44^lo^, and/or CD62L^hi^) CD4^+^ T lymphocytes can induce severe colitis and inflammation in other organs when transferred into immunodeficient mice (*25*–*27*). In the case of MP CD4^+^ T lymphocytes, a recent report demonstrated that CCR6^hi^ MP cells can exaggerate neuroinflammation triggered by myelin oligodendrocyte glycoprotein–specific naïve CD4^+^ T cells in an innate manner (*14*). However, immunological behaviors of MP CD4^+^ T lymphocytes and especially their T-bet^−^ Rorγt^−^ subset in lymphopenic environments have not been defined.

In addition to the presumptive inflammatogenic nature of MP CD4^+^ T lymphocytes, it is also possible that the same cells share immunosuppressive function with regulatory T (T_reg_) cells since these two types of cells have TCRs with relatively high affinity to self-antigens. We previously reported that naïve precursors with higher TCR affinity to self-antigens can more efficiently generate MP cells in the periphery (*6*), whereas in the thymus, relatively strong TCR signals (below the threshold for negative selection) instruct thymocytes to differentiate into thymic T_reg_ (tT_reg_) cells (*28*, *29*). These observations suggest some relationship between MP CD4^+^ T lymphocytes and T_reg_ cells.

In this study, we have examined the behaviors of MP CD4^+^ T lymphocytes and, in particular, their T-bet^−^ Rorγt^−^ subset in lymphopenic and inflammatory settings. Our observations identify naturally arising T-bet^−^ Rorγt^−^ MP CD4^+^ T lymphocytes as a polyclonal, transcriptomically immature “undifferentiated” precursor that can rapidly proliferate to give rise to T_H_1, T_H_17, and T_reg_ cells to contribute to pathogenesis of inflammation.

## RESULTS

### MP CD4^+^ T lymphocytes accumulate in multiple organs including colon and lungs to induce inflammatory responses in a lymphopenic environment

As a first step to examine the immunological function of T-bet^−^ Rorγt^−^ MP CD4^+^ T lymphocytes, we sought to define the behavior of MP cells as a whole population in lymphopenic environments. Because it is well known that CD45RB^hi^ but not CD45RB^lo^ CD4^+^ T cells can induce severe colitis in lymphodeficient mice (*25*), we first analyzed the composition of these two CD4^+^ T lymphocyte populations. While essentially all CD45RB^hi^ cells were naïve, CD45RB^lo^ cells comprised a mixture of CD44^lo^ CD62L^hi^ Foxp3^−^ naïve, CD44^hi^ CD62L^lo^ Foxp3^−^ MP, and Foxp3^+^ T_reg_ cells (fig. S1), indicating that CD45RB is not useful as a sole marker for distinguishing MP from naïve and T_reg_ cells.

**Fig. 1.**
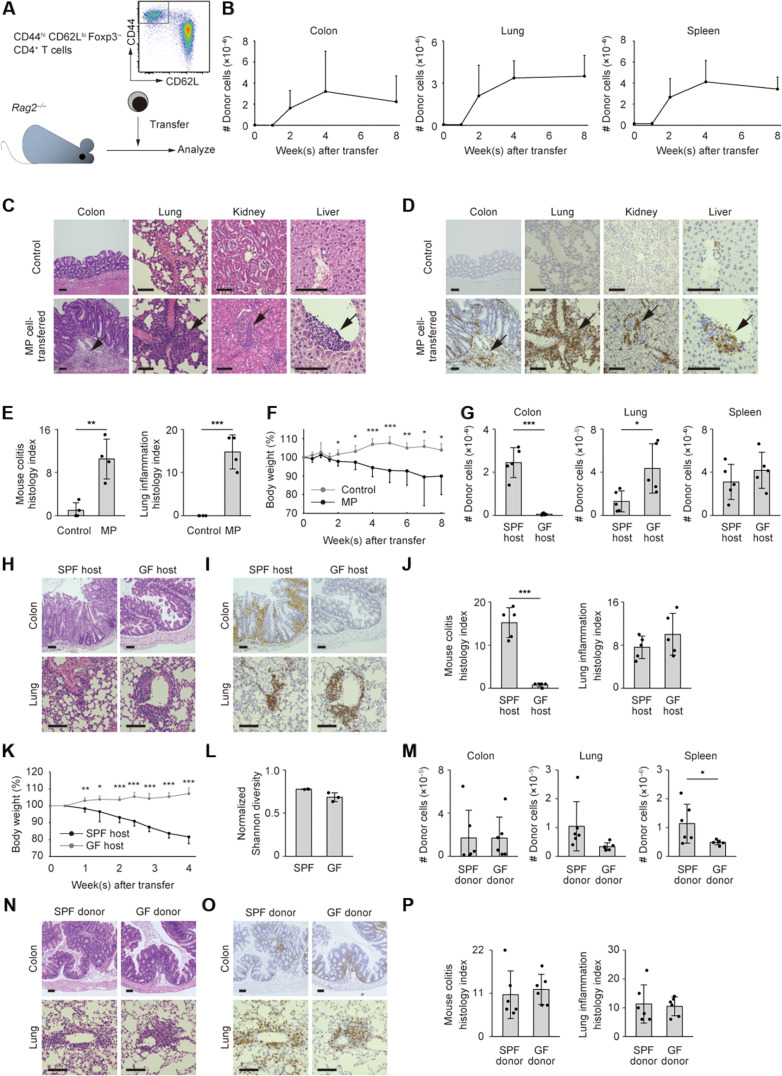
Self-reactive MP cells accumulate in multiple organs of *Rag2^−/−^* mice. (**A**) Experimental design. *Rag2*^−/−^ mice received intravenous injection of MP cells (5 × 10^5^ cells per host) and were analyzed at different time points. (**B**) The number of donor cells accumulating in the indicated organs of *Rag2*^−/−^ mice (*n* = 4). (**C** to **E**) In the above experiments, histological analyses using H&E and immunohistochemical staining were performed 4 weeks after transfer. For the latter staining, CD4^+^ cells were detected by CD4 mAb and secondary peroxidase-conjugated antibody. Representative images display H&E and immunohistochemical staining, while the bar graphs indicate histological scores of colitis and lung inflammation (*n* = 4). Pooled from two independent experiments. (**F**) Relative body weight at the indicated time points (*n* = 5 to 6). Pooled from three independent experiments. (**G**) The number of SPF MP donor cells accumulating in the indicated organs of SPF versus GF *Rag1*^−/−^ mice 4 weeks after transfer (*n* = 5). (**H** to **J**) In the above experiments, histological analyses were performed. Representative images depict H&E and CD4-directed immunohistochemical staining of the indicated organs, while the bar graphs show histological scores of inflammation (*n* = 5). (**K**) Relative body weight of SPF versus GF *Rag1*^−/−^ mice transferred with SPF MP cells (*n* = 5). Representative of two experiments. (**L**) TCRβ CDR3 regions were sequenced in MP cells obtained from unchallenged SPF and GF mice. The bar graph shows the normalized Shannon index (*n* = 3). (**M**) The number of SPF versus GF MP donor cells accumulating in the indicated organs of SPF *Rag1*^−/−^ hosts 4 weeks after transfer (*n* = 6). (**N** to **P**) H&E and CD4-directed immunohistochemical staining of the indicated sections together with bar graphs showing histological scores (*n* = 6). Pooled from two independent experiments. Graphs: means ± SD, each symbol represents an individual mouse. Scale bar, 100 μm. *P < 0.05, **P < 0.01, ***P < 0.001.

We thus sorted for Foxp3^−^ CD44^hi^ CD62L^lo^ MP CD4^+^ T lymphocytes from Foxp3-red fluorescent protein (RFP) reporter mice and transferred these cells into *Rag2^−/−^* animals (Fig. 1A). Flow cytometric analyses at different time points showed that donor cells accumulated in the colon, lungs, and spleen with time and reached a plateau in number approximately 4 weeks after transfer (Fig. 1B; gating strategy is described in Materials and Methods). Intravascular staining with fluorescein isothiocyanate (FITC)–conjugated antibody revealed that a vast majority of donor cells detected in the lungs represented those that infiltrated into lung tissues rather than those circulating in the blood (fig. S2). Consistent with this, immunohistochemical together with hematoxylin and eosin (H&E) staining in adjacent sections revealed that CD4^+^ donor cells infiltrated into the inflamed portions in lamina propria as well as submucosal tissues of the colon, interstitial, and, in particular, perivascular regions of lungs, interstitial area of kidneys, and Glison’s sheaths of the liver (Fig. 1, C and D). Histological indexes of colitis and lung inflammation were significantly higher in MP-transferred as compared to control groups (Fig. 1E). Furthermore, clinical symptoms of colitis, lowered blood oxygen saturation, and elevated levels of blood urine nitrogen (BUN) and transaminases (AST and ALT) in serum were observed in *Rag2*^−/−^ mice that had received MP cells (fig. S3), suggesting malfunction of colon, lungs, kidneys, and liver. As a result, body weight was significantly reduced in MP cell–transferred animals (Fig. 1F). Together, these findings suggest that when transferred into *Rag2*^−/−^ mice, MP cells as a whole population accumulate in multiple organs including colon and lungs to induce inflammatory responses.

In the case of naïve cell–induced colitis, commensal microbiota of host origins play an essential role in the development of the disease (*30*). To ask whether MP cells can trigger inflammation dependently or independently of host-derived commensal microbiota, we transferred MP cells derived from SPF animals into SPF versus GF *Rag1^−/−^* mice. As expected, donor cell accumulation in the colon was eliminated in GF hosts (Fig. 1, G to J). On the other hand, donor cell accumulation in lungs or spleen was not decreased (Fig. 1, G to J). Body weight reduction was inhibited in GF animals (Fig. 1K). Thus, host-derived commensal microbiota are essential for MP cell accumulation in the gut but not the lungs. These results also suggest that body weight reduction of the host animals mainly reflect the degree of MP cell immune responses in the gut but not lungs.

We next asked whether MP cell accumulation in the colon and lungs is affected by the presence of commensal microbiota of donor mouse origins. We previously reported that the total number of MP cells in secondary lymphoid organs under steady state is essentially the same between SPF and GF mice (*4*, *5*). In the present study, we further compared the TCR repertoire diversity of MP cells obtained from unchallenged SPF versus GF animals by performing TCR deep sequencing and found no significant difference (Fig. 1L), suggesting that commensal microbiota do not affect the development of MP cells present at homeostasis, either in number or in clonal diversity.

To determine whether MP cells that have developed in the absence of donor-derived commensal antigens can induce the above immune responses, we further transferred MP cells from SPF versus GF donor mice into SPF *Rag1^−/−^* animals. As shown in Fig. 1 (M to P), MP cells derived from SPF and GF mice equally accumulated in the colon and lungs, although infiltration of GF MP cells to the spleen was slightly reduced. Therefore, MP cells that have been originally generated independently of commensal antigens can accumulate in the intestine and lungs of lymphopenic animals, suggesting an essential role for self-antigen–driven MP cells in the induction of immune responses in these organs.

### IFN-γ and IL-17A cooperatively or compensatorily contribute to MP cell–driven immune responses in the colon but not lungs

**Fig. 2.**
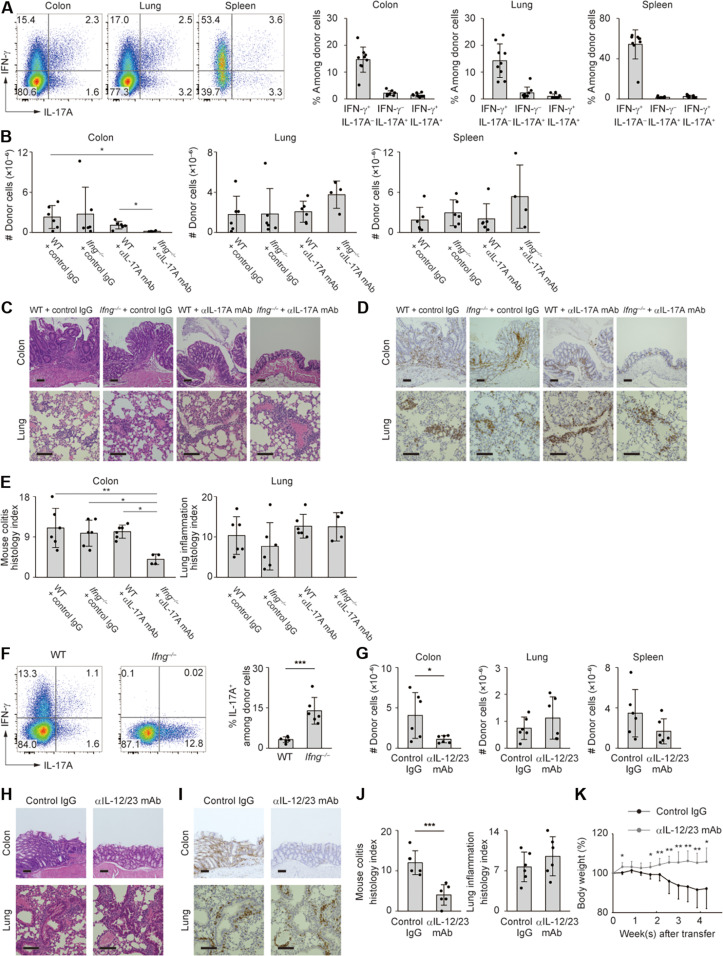
IFN-γ and IL-17A play redundant roles in lymphopenia-induced MP cell responses in the intestine but not lungs. (**A**) MP cells were intravenously transferred into *Rag2*^−/−^ mice and analyzed in the indicated organs 4 weeks later. Cells were incubated ex vivo in the presence of phorbol 12-myristate 13-acetate and ionomycin for 4 hours. The dot plots display expression of IFN-γ and IL-17A, while the bar graphs show the frequency of cytokine-producing cells among the total donor population (*n* = 9). Pooled from three independent experiments. (**B** to **E**) *Rag2^−/−^* animals received MP cells of WT or *Ifng^−/−^* origins and were subsequently administered anti–IL-17A mAb or control immunoglobulin G (IgG). (B) The bar graphs show the number of donor cells from each group in the indicated organs (*n* = 4 to 6). (C to E) Representative images of H&E and CD4-directed immunohistochemical staining together with bar graphs indicating histological scores (*n* = 4 to 6). Pooled from three independent experiments. (**F**) WT versus *Ifng^−/−^* MP cells were transferred into *Rag2*^−/−^ mice and measured for their cytokine expression in the colon 4 weeks later. The dot plots display expression of cytokines, while the bar graph indicates the frequency of IL-17A^+^ cells among the total donor population (*n* = 6). Pooled from two independent experiments. (**G** to **J**) *Rag2*^−/−^ mice that had received MP cells were treated with anti–IL-12/23 p40 mAb or control IgG and the donor cells analyzed 4 weeks later. (G) The bar graphs show the number of donor cells in the indicated organs (*n* = 6). (H to J) Representative images displaying H&E and CD4-directed immunohistochemical staining together with bar graphs indicating histological scores are shown (*n* = 6). (**K**) The graph shows relative body weight of *Rag2^−/−^* mice from each group (*n* = 6). Data are pooled from two independent experiments. Graphs: means ± SD, each symbol represents an individual mouse. Scale bar, 100 μm. *P < 0.05, **P < 0.01, ***P < 0.001.

Because T_H_1 and T_H_17 contribute to pathogenesis of colitis driven by naïve CD4^+^ T lymphocytes (*31*, *32*), we sought to clarify the roles for cytokines IFN-γ and IL-17A in lymphopenia-induced immune responses of MP cells. For this purpose, we examined their expression levels in MP-derived donor cells transferred into *Rag2*^−/−^ mice. Four weeks after transfer, MP donor cells produced IFN-γ as well as IL-17A (Fig. 2A), suggesting that MP CD4^+^ T lymphocytes can generate T_H_1 and, to a lesser extent, T_H_17 responses in a lymphopenic environment.

To clarify the roles for IFN-γ and IL-17A in the MP cell response, we transferred MP cells obtained from wild-type (WT), *Ifng*^−/−^, or *Il1*7*a*^−/−^ mice into *Rag2*^−/−^ hosts. WT and *Ifng*^−/−^ MP cells equally accumulated in the intestine, lungs, and spleen (Fig. 2, B to E). Similarly, WT and *Il17a*^−/−^ MP lymphocytes accumulated in these organs to the same degree (fig. S4, A to D). Consistent with this, treatment with anti–IFN-γ or anti–IL-17A monoclonal antibodies (mAbs) to *Rag2*^−/−^ mice that had received WT MP cells did not ameliorate histological inflammation in the colon or lungs, although the former mAb significantly inhibited donor cell accumulation in the spleen (Fig. 2, B to E, and fig. S5). These results make the possibility unlikely that either IFN-γ or IL-17A alone derived from MP donor cells plays an essential role in the induction of intestinal and lung inflammation.

Because *Ifng*^−/−^ MP donor cells produced higher levels of IL-17A in *Rag2*^−/−^ hosts (Fig. 2F), we hypothesized that IFN-γ and IL-17A play cooperative or compensatory roles in MP cell accumulation and inflammatory responses. To test this, we transferred *Ifng*^−/−^ MP cells into *Rag2*^−/−^ mice that were subsequently subjected to anti–IL-17A mAb treatment. As shown in Fig. 2, B and E, donor cell accumulation in the colon but not lungs was significantly reduced in *Rag2*^−/−^ animals that received *Ifng*^−/−^ MP cells when IL-17A was blocked. This finding suggests that IFN-γ and IL-17A cooperatively or compensatorily contribute to MP cell–driven immune responses in the gut but not lungs under lymphopenic settings.

Production of IFN-γ and IL-17A by helper T lymphocytes as well as ILCs is promoted by cytokines IL-12 and IL-23, respectively (*33*). Because IL-12 and IL-23 share a common subunit p40 (*34*, *35*), blockade of p40 can inhibit the activities of both of these cytokines (*36*), and anti–IL-12/23 p40 mAb has been shown to be effective for treatment of intestinal inflammation in both mice (*37*, *38*) and humans (*39*, *40*). To ask whether blockade of IL-12/23 inhibits MP cell–driven inflammation, we administered anti–IL-12/23 p40 mAb to *Rag2*^−/−^ mice that had received MP cells. Antibody treatment significantly inhibited accumulation of T_H_1/17 donor cells in the colon but not lungs (Fig. 2, G to J, and fig. S4E). Consistent with this, body weight reduction was significantly inhibited by the antibody treatment (Fig. 2K). These results argue that lymphopenia-induced MP cell responses in the intestine and lungs are governed by distinct mechanisms; the former requires IFN-γ, IL-17A, and IL-12/23 for its induction, while the latter is less dependent on these cytokines.

### MP cells with higher TCR affinity to self-antigens produce larger amounts of IFN-γ

**Fig. 3.**
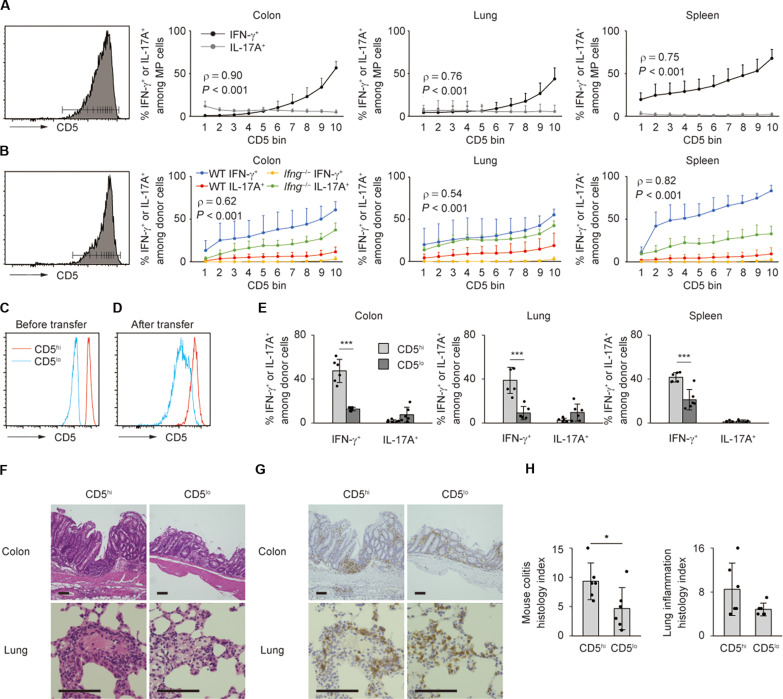
TCR affinity of MP CD4^+^ T lymphocytes to self-antigens is positively associated with their capacity to produce inflammatory cytokines. (**A** and **B**) MP cells were measured for their CD5 and cytokine expression levels at homeostasis in (A) whereas transferred into *Rag2*^−/−^ mice and reanalyzed for these parameters 4 weeks later in (B). The histograms define 10% bins of CD5 expression in MP cells, while the graphs show the frequency of IFN-γ^+^ and IL-17A^+^ fractions among MP cells of each CD5 bin (*n* = 6). Pooled from two independent experiments. (**C** and **D**) Sorted CD5^hi^ and CD5^lo^ MP cells were individually transferred to *Rag2*^−/−^ mice, and their CD5 levels reanalyzed 4 weeks later. The histograms depict CD5 expression in each group (C) before and (D) after transfer. Representative data from two independent experiments. (**E**) The bar graphs show the frequency of IFN-γ^+^ and IL-17A^+^ cells among each donor cell population from the above experiments (*n* = 6). (**F** to **H**) Representative images displaying H&E and CD4-directed immunohistochemical staining together with bar graphs indicating histological scores are shown (*n* = 6). Pooled from two independent experiments. Graphs: means ± SD, each symbol represents an individual mouse. Scale bar, 100 μm. *P < 0.05, ***P < 0.001.

The aforementioned results show that self-driven MP cells can respond to available antigens including commensal antigens and accumulate in the gut in a cytokine-dependent manner (Figs. 1 and 2). Given that T lymphocytes with higher TCR affinity to self have higher affinity to foreign antigens as well (*41*, *42*), we hypothesized that there is a correlation between the strength of TCR affinity to self-antigens in MP cells and their capacity to produce cytokines. To test this possibility, we plotted the frequency of IFN-γ^+^ as well as IL-17A^+^ fraction against the strength of TCR affinity to self that is reflected by CD5 levels in MP CD4^+^ T lymphocytes present in steady state. As shown in Fig. 3A, MP cells with higher CD5 expression exhibited augmented IFN-γ levels with the IL-17A production largely unchanged, suggesting positive correlation between CD5 levels on MP cells and their capacity to produce IFN-γ. To confirm this finding in lymphopenic environments, we next transferred MP cells of WT and *Ifng*^−/−^ origins into *Rag2*^−/−^ mice. Four weeks later, WT donor cells with higher CD5 expression produced elevated IFN-γ but not IL-17A, with the latter cytokine production being promoted in *Ifng*^−/−^ donor cells (Fig. 3B). These data further support the above concept under lymphopenic conditions as well.

The above results suggested that WT MP cells with higher TCR affinity to self-antigens can produce larger amounts of IFN-γ. To formally test this hypothesis, we further sorted for CD5^hi^ and CD5^lo^ MP cells and transferred these populations separately into *Rag2^−/−^* mice. Relative expression levels of CD5 were maintained before and after transfer (Fig. 3, C and D). As shown in Fig. 3E, CD5^hi^ MP cells generated greater IFN-γ but not IL-17A responses. Consistent with this observation, CD5^hi^ MP cells induced more severe histological colitis than did their CD5^lo^ counterparts, whereas lung inflammation was unchanged between the two groups (Fig. 3, F to H). Together, these data support a model in which MP cells with higher TCR affinity to self more strongly respond to available antigens including commensal antigens to more efficiently produce inflammatory cytokines, thereby contributing to their immune responses in the gut.

### MP as compared to naïve CD4^+^ T cells more efficiently generate T_reg_ cells in a TGFβ-dependent manner

**Fig. 4.**
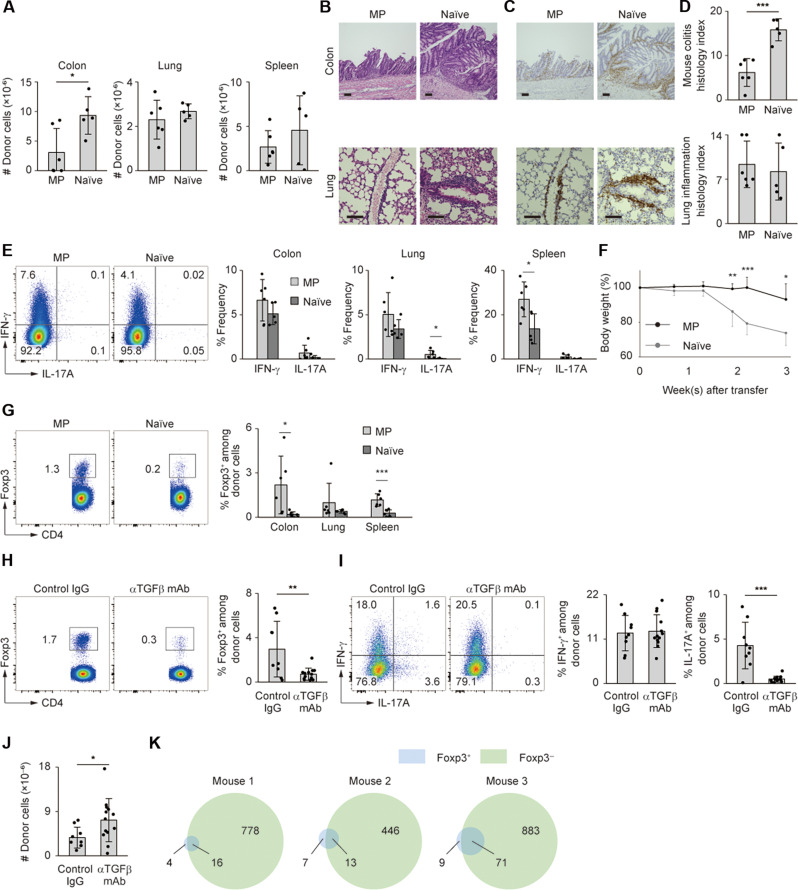
MP versus naïve cells more efficiently differentiate into T_reg_ cells in *Rag2^−/−^* mice. (**A** to **E**) Sorted MP or naïve CD4^+^ T lymphocytes were transferred into *Rag2*^−/−^ mice and the donor cells analyzed 3 weeks later. (A) The bar graphs show the number of donor cells accumulating in the indicated organs (*n* = 5 to 6). (B to D) Representative images of H&E and CD4-directed immunohistochemical staining together with bar graphs showing histological scores are displayed (*n* = 5 to 6). (E) The bar graphs show the frequency of IFN-γ^+^ and IL-17A^+^ cells among the total donor populations in the indicated organs (*n* = 5 to 6). Pooled from two independent experiments. (**F**) Relative body weight of *Rag2^−/−^* mice from each group in the above experiments (*n* = 6). Pooled from two independent experiments. (**G**) The representative plots show Foxp3 expression in colonic donor cells, while the bar graph indicates the frequency of Foxp3^+^ cells among the total donor populations in the indicated organs (*n* = 6 to 7). Pooled from two independent experiments. (**H** to **J**) Anti-TGFβ mAb or control IgG were administered to *Rag2*^−/−^ mice that had received MP cells, and donor cells analyzed 4 weeks later. (H and I) The representative plots show expression of Foxp3, IFN-γ, and IL-17A in colonic donor cells, whereas the bar graphs indicate the frequency of Foxp3^+^, IFN-γ^+^, and IL-17A^+^ cells among the total donor population (*n* = 9 to 13). (J) A graph showing the total number of donor cells accumulating in the gut (*n* = 9 to 13). Pooled from three independent experiments. (**K**) The Venn diagrams display TCR clonotypes of Foxp3^−^ and Foxp3^+^ donor cells accumulating in the intestine of *Rag2*^−/−^ mice. The numbers show those of TCR clonotypes detected in Foxp3^−^ donor population only, Foxp3^+^ donor population only, and both Foxp3^−^ and Foxp3^+^ donor populations. Graphs: means ± SD, each symbol represents an individual mouse. Scale bar, 100 μm. *P < 0.05, **P < 0.01, ***P < 0.001.

We next compared lymphopenia-induced immune responses of MP versus naïve cells by separately transferring MP and naïve Foxp3^−^ CD4^+^ T lymphocytes into *Rag2*^−/−^ mice. As shown in Fig. 4 (A to D), MP as compared to naïve cells less efficiently dwelled in the colon, whereas both types of donor cells equally accumulated in lungs and spleen. Regarding the capacity of donor cells to produce cytokines, difference in IFN-γ and IL-17A expression levels was, if any, minimal between the two types of donor cell populations (Fig. 4E). As a result, body weight reduction was milder in the MP cell–transferred group (Fig. 4F). Thus, MP CD4^+^ T lymphocytes generate a milder immune response in the colon than do naïve cells.

To clarify the mechanism that governs the above difference between MP and naïve CD4^+^ T lymphocytes, we examined Foxp3 expression on donor cells. MP cells more efficiently gave rise to the Foxp3^+^ subset than did naïve cells, especially in the gut (Fig. 4G), suggesting that MP cells are more prone than naïve CD4^+^ T lymphocytes to T_reg_ differentiation.

In the case of peripheral T_reg_ (pT_reg_) cell development from naïve precursors, transforming growth factor–β (TGFβ) plays a key role (*43*–*46*). To ask whether TGFβ is also important for T_reg_ development from MP cells, we administered anti-TGFβ antibody to *Rag2^−/−^* mice that had received MP cells. The antibody treatment significantly reduced T_reg_ and T_H_17 but not T_H_1 cells and increased the total number of donor cells accumulating in the colon (Fig. 4, H to J). T_reg_ development from MP cells is therefore promoted by TGFβ in lymphopenic settings.

The aforementioned results suggest that some MP CD4^+^ T lymphocytes can convert to T_H_1/17 and T_reg_ cells in response to IL-12/23 and TGFβ, respectively, in the colon. To determine whether there are common MP cell clones capable of generating both effector and T_reg_ cells, we transferred Foxp3^−^ MP cells into *Rag2*^−/−^ mice and performed TCR sequencing of CDR3 regions in Foxp3^−^ as well as Foxp3^+^ donor cells accumulating in the gut. As shown in Fig. 4K, TCRs of Foxp3^+^ donor cells overlapped with those of their Foxp3^−^ counterparts, arguing that there are some MP cell clones that can generate both effector and T_reg_ cells.

### MP-derived T_reg_ cells inhibit MP-driven immune responses

**Fig. 5.**
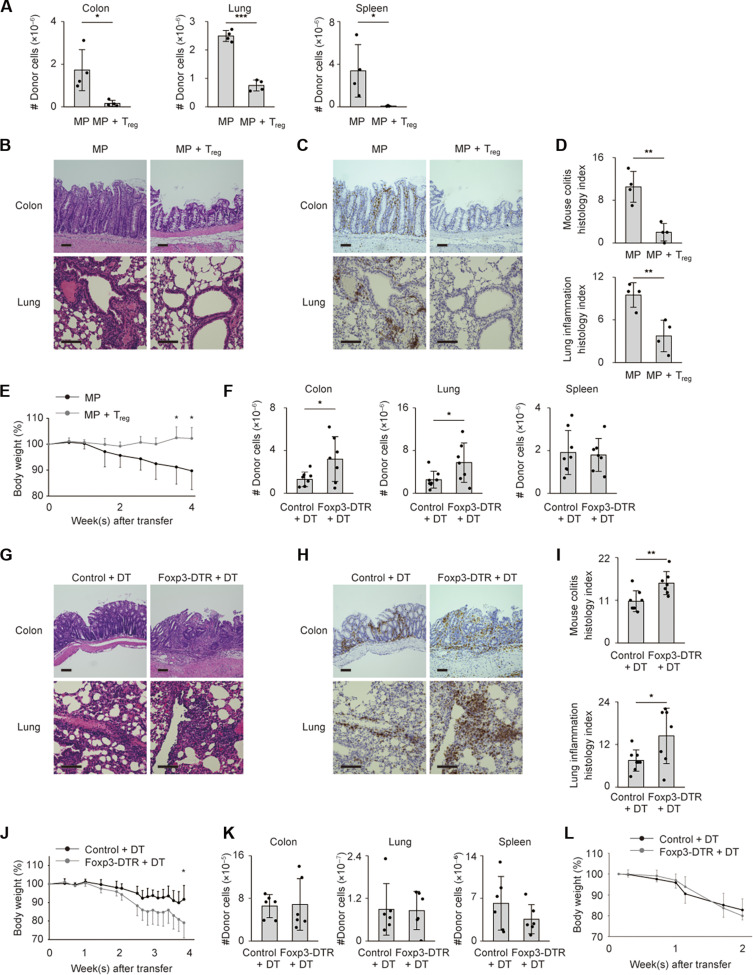
Lymphopenia-induced MP cell responses are suppressed by MP-derived T_reg_ cells. (**A** to **E**) Sorted MP CD4^+^ T lymphocytes were transferred into *Rag2*^−/−^ animals with or without T_reg_ cells and analyzed 4 weeks later. (A) Graphs showing the number of MP donor cells accumulating in the indicated organs (*n* = 4). (B to D) Representative images of H&E and CD4-directed immunohistochemical staining of the large intestinal and lung sections together with bar graphs showing histological quantitation (*n* = 4). (E) A graph indicating the relative body weight of *Rag2^−/−^* hosts from each group at different time points (*n* = 4). Pooled from two independent experiments. (**F** to **J**) MP cells obtained from Foxp3-DTR or control Foxp3-RFP mice had been transferred into *Rag2*^−/−^ animals that were subsequently administered DT over the course of the experiments. (F) Graphs showing the number of donor cells accumulating in the indicated organs (*n* = 7 to 8). (G to I) H&E and CD4-directed immunohistochemical staining of large intestinal and lung sections together with bar graphs showing histological quantitation (*n* = 7 to 8). Pooled from three independent experiments. (J) A graph indicating the relative body weight of *Rag2^−/−^* hosts from each group (*n* = 5). Pooled from two independent experiments. (**K** and **L**) Naïve cells from Foxp3-DTR or control Foxp3-RFP mice were transferred into *Rag2*^−/−^ mice that were then subjected to DT treatment. (K) The bar graphs show the number of donor cells accumulating in the indicated organs (*n* = 6). (L) The line graph displays the relative body weight of the host animals from each group (*n* = 6). Pooled from two independent experiments. Graphs: means ± SD, each symbol represents an individual mouse. Scale bar, 100 μm. *P < 0.05, **P < 0.01, ***P < 0.001.

We sought to determine whether T_reg_ cells inhibit MP cell immune responses triggered in *Rag2*^−/−^ mice. For this purpose we transferred MP cells with or without T_reg_ cells into *Rag2^−/−^* mice. Cotransfer of T_reg_ cells significantly inhibited donor cell accumulation in the colon, lungs, and spleen and body weight reduction (Fig. 5, A to E), showing that MP cell accumulation in the gut and lungs can be inhibited by cotransfer of T_reg_ cells similarly to naïve cell-induced colitis.

To further determine whether T_reg_ cells that have differentiated from MP cells can inhibit MP cell–driven immune responses, we transferred Foxp3^−^ MP cells sorted from Foxp3-diphtheria toxin receptor-transgenic (Foxp3-DTR) mice into *Rag2*^−/−^ hosts that were subsequently treated with diphtheria toxin (DT). Depletion of Foxp3^+^ cells augmented MP cell accumulation in the colon and lungs and exaggerated body weight loss (Fig. 5, F to J). On the other hand, when Foxp3^−^ naïve donor cells were obtained from Foxp3-DTR mice and similar experiments to the above performed, ablation of T_reg_ cells did not significantly exaggerate naïve cell–driven immune responses (Fig. 5, K and L). Together, these data demonstrate that MP-derived T_reg_ cells can inhibit lymphopenia-induced immune responses of MP cells.

### MP cells contain a polyclonal, undifferentiated subpopulation at homeostasis

**Fig. 6.**
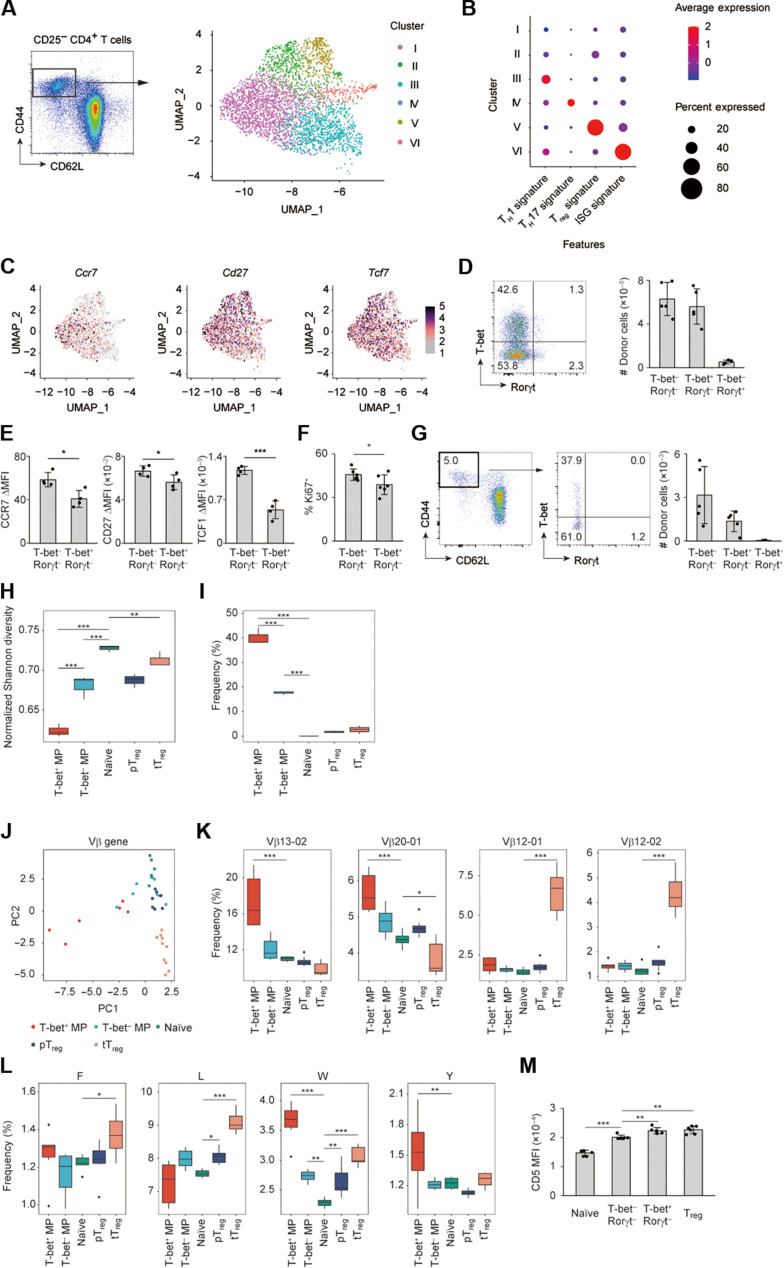
MP CD4^+^ T lymphocytes contain a polyclonal, undifferentiated subpopulation. (**A** to **C**) Single-cell RNA sequencing of CD44^hi^ CD62L^lo^ CD25^−^ CD4^+^ αβT cells. (A) The plot displays single cells determined by the UMAP algorithm. (B) The dot plot displays both relative expression of each signature-associated genes and the percentage of each score^hi^ cells. (C) Expression levels of the indicated genes in each cell are overlaid on the UMAP plot. (**D**) The dot plot displays expression of T-bet and Rorγt in Foxp3^−^ MP cells from T-bet/Rorγt/Foxp3 reporter mice, whereas the bar graph shows the number of the indicated MP subpopulations (*n* = 5). (**E**) The ΔMFI of CCR7, CD27, and TCF1 levels in the indicated populations (*n* = 4), calculated by subtracting MFI of nonstaining control from that of each population. (**F**) The frequency of Ki67^+^ cells among MP subpopulations (*n* = 6). Representative of two independent experiments. (**G**) Naïve CD4^+^ T cells from T-bet/Rorγt/Foxp3 reporter mice were transferred into WT mice and donor-derived MP cells analyzed 10 days later. The plots display expression of CD44, CD62L, T-bet, and Rorγt in donor cells, whereas the graph shows the number of the indicated MP donor subsets (*n* = 5). Representative of two independent experiments. (**H** and **I**) The box plots show (H) normalized Shannon indexes of TCRβ sequences derived from the indicated populations and (I) the frequency of clonally expanding clones in the indicated populations (*n* = 3). (**J** and **K**) A PCA plot displaying the Vβ profiles of the indicated populations together with box plots showing the frequency of each Vβ-expressing clones (*n* = 6 to 10). (**L**) Box plots presenting the contents of the indicated amino acids among the CDR3 region (*n* = 6 to 10). (**M**) The MFI of CD5 in the indicated populations at homeostasis (*n* = 6). Representative of two independent experiments. Bar graphs: means ± SD, each symbol represents an individual mouse. *P < 0.05, **P < 0.01, ***P < 0.001.

On the basis of the aforementioned observations that MP CD4^+^ T lymphocytes can give rise to the functional T_H_1, T_H_17, and T_reg_ subsets in *Rag2*^−/−^ animals, we next sought to determine whether MP cells comprise heterogeneous subpopulations at homeostasis, and, if so, which subpopulation exerts this unique immune function. We previously obtained single cell–level gene expression profiles of CD44^hi^ CD62L^lo^ CD25^−^ CD4^+^ αβT cells present in steady state (*5*), which represent a mixture of MP CD4^+^ T lymphocytes together with CD25^−^ Foxp3^+^ T_reg_ cells (*47*). Using the same data, we reanalyzed the data by the Uniform Manifold Approximation and Projection (UMAP) algorithm and performed clustering analysis. As shown in Fig. 6 (A and B) and fig. S6A, the cells consisted of multiple clusters with distinct gene expression signatures. Specifically, MP CD4^+^ T cells contained two undifferentiated clusters that did not express known lineage-determining transcription factors (LDTFs) (clusters I and II). Moreover, we found major T_H_1-like (cluster III) and minor T_H_17-like (cluster IV) fractions that are adjacent to each other. In addition, cells with high *Foxp3* expression representing CD25^−^ Foxp3^+^ T_reg_ cells (cluster V) and those expressing IFN-stimulated genes (ISGs) (cluster VI) were present. Furthermore, when expression of naïve/memory cell–related genes was examined in each cluster, MP cells and, especially, their clusters I and II expressed high levels of *Ccr7*, *Cd27*, and *Tcf7* (Fig. 6C). Thus, in addition to major T_H_1-like and minor T_H_17-like subsets, MP cells contain transcriptomically undifferentiated subpopulations at homeostasis.

We sought to validate the above results by flow cytometry. To do so, we used T-bet/Rorγt/Foxp3 triple reporter mice and analyzed T-bet and Rorγt expression in Foxp3^−^ MP CD4^+^ T lymphocytes. As shown in Fig. 6D, Foxp3^−^ MP cells consisted of major T-bet^−^ Rorγt^−^ and T-bet^+^ Rorγt^−^ subpopulations as well as a minor fraction of Rorγt^+^ cells. Moreover, T-bet^−^ Rorγt^−^ MP cells expressed higher levels of CCR7, CD27, and TCF1 than did their T-bet^+^ Rorγt^−^ counterparts (Fig. 6E). Furthermore, the former subset expressed higher levels of a cell cycle marker Ki67 than did the latter (Fig. 6F). These data phenotypically confirm the undifferentiated features of T-bet^−^ Rorγt^−^ MP cells.

On the basis of our previous finding that MP cells are generated from peripheral naïve CD4^+^ T lymphocytes in steady state (*6*), we asked whether each of the MP subsets can be generated from naïve cells. To do so, we transferred naïve CD4^+^ T lymphocytes obtained from T-bet/Rorγt/Foxp3 reporter mice into congenic WT mice and examined reporter expression of donor-derived MP cells 10 days later. As shown in Fig. 6G, newly generated MP cells comprised T-bet^−^ Rorγt^−^ and T-bet^+^ Rorγt^−^ subpopulations, whereas the Rorγt^+^ subset was hardly detected. Thus, undifferentiated MP cells can be directly generated from naïve precursors at homeostasis.

We further characterized undifferentiated versus T_H_1-differentiated MP cells at a TCR sequence level. For this purpose, TCR sequences were compared among T-bet^+^ Rorγt^−^ MP, T-bet^−^ Rorγt^−^ MP, naïve, pT_reg_, and tT_reg_ cells present in steady state. As shown in Fig. 6H, the TCR repertoire of T-bet^−^ MP cells was more diverse than that of their T-bet^+^ counterparts, whereas naïve cells were most polyclonal. Consistent with this, clonally expanding clones that occupied >0.1% of the total T cell subpopulation accounted for significantly lower fraction of T-bet^−^ versus T-bet^+^ MP cells (Fig. 6I). These data argue that while both T-bet^−^ and T-bet^+^ MP cells are less diverse than naïve CD4^+^ T lymphocytes in terms of TCR repertoire, the degree of clonal expansion is substantially lower in the former MP subset.

We also compared TCR Vβ usage of each T cell subpopulation. A principal components analysis (PCA) showed that the Vβ usage pattern of T-bet^+^ MP cells was distinct from that of naïve and tT_reg_ cells (Fig. 6J). Specifically, Vβ13-02 and Vβ20-01 were enriched in T-bet^+^ MP cells, whereas Vβ12-01 and Vβ12-02 frequently detected in tT_reg_ cells (Fig. 6K). In these data, the Vβ usage pattern of T-bet^−^ MP cells was close to that of naïve rather than T-bet^+^ MP or tT_reg_ cells (Fig. 6, J and K). These results demonstrate that while certain types of TCR Vβ genes are enriched in T-bet^+^ MP and tT_reg_ cells as compared to naïve CD4^+^ T lymphocytes at homeostasis, the usage pattern of the same genes in T-bet^−^ MP cells is similar to that in naïve cells.

On the basis of previous reports showing that hydrophobic amino acid residues in the middle CDR3 region promote the development of self-reactive T cell clones (*48*, *49*), we further measured the quantity of each hydrophobic amino acid in the same region of TCRβ derived from T cell subpopulations. Phenylalanine (F), leucine (L), and tryptophan (W) were enriched in tT_reg_ cells, whereas W and tyrosine (Y) were frequently detected in T-bet^+^ MP cells (Fig. 6L), consistent with the notion that these two types of CD4^+^ T cells have high TCR affinity to self-antigens (*6*, *29*). On the other hand, such enrichment of hydrophobic amino acids was, if any, minimal in T-bet^−^ MP cells (Fig. 6L). Consistent with this, CD5 levels were highest in T_reg_ and T-bet^+^ MP cells whereas lowest in naïve cells, with those of the T-bet^−^ MP subset in between (Fig. 6M). Together, these data argue that while highly self-reactive T cell clones are enriched in T_reg_ and T-bet^+^ MP cells, T-bet^−^ MP cells represent a clonally less selected subpopulation in terms of TCR affinity to self-antigens.

### The undifferentiated MP subset has a potential to generate T_H_1, T_H_17, and T_reg_ cells

**Fig. 7.**
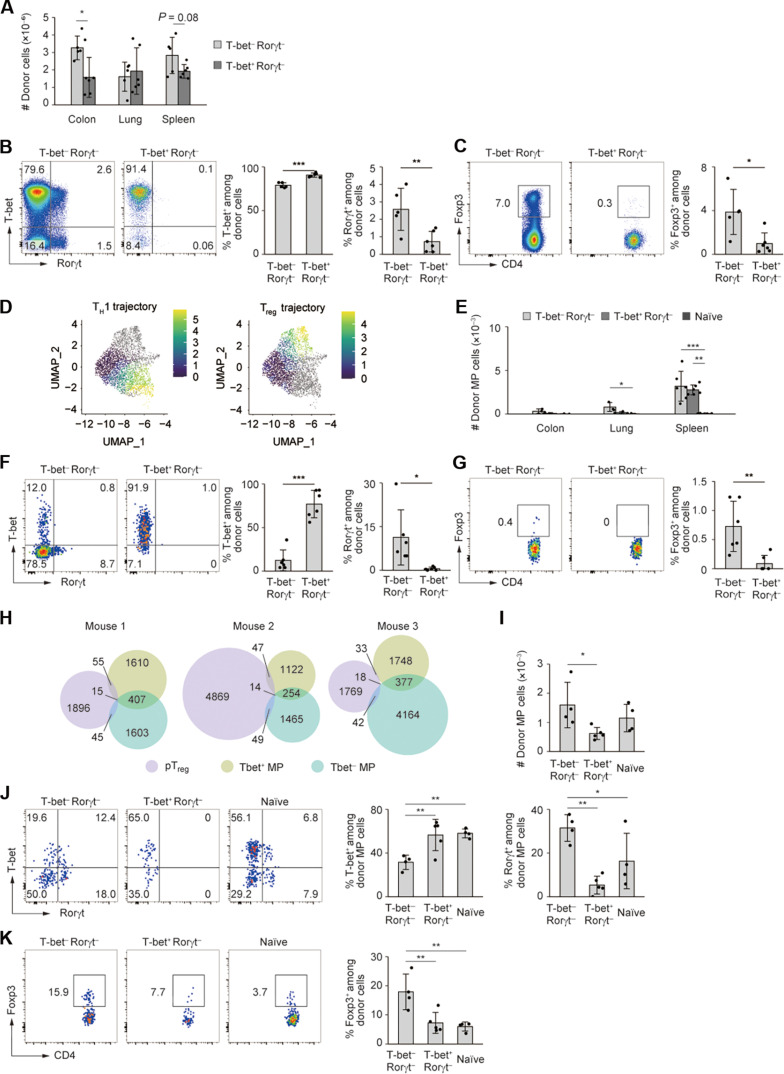
Undifferentiated MP cells can generate T-bet^+^, Rorγt^+^, and Foxp3^+^ subsets. (**A** to **C**) T-bet^−^ Rorγt^−^ and T-bet^+^ Rorγt^−^ MP cells were sorted from T-bet/Rorγt/Foxp3 reporter mice and separately transferred into *Rag2*^−/−^ hosts. (A) The bar graph indicates the number of donor cells accumulating in the indicated organs 4 weeks later (*n* = 5 to 6). (B and C) The dot plots depict reporter expression in colonic donor cells, whereas the bar graphs indicate the frequency of each reporter-positive fraction (*n* = 5 to 6). Pooled from two independent experiments. (**D**) In Fig. 6A, T_H_1 and T_reg_ trajectories (clusters I > III and I > II, respectively) were analyzed. Pseudotime for each trajectory is projected onto the UMAP. (**E** to **G**) T-bet^−^ Rorγt^−^ MP, T-bet^+^ Rorγt^−^ MP, or naïve cells were transferred into CD45.1 WT mice and the donor cells analyzed 10 days later. (E) A bar graph showing the number of donor-derived MP cells accumulating in the indicated organs (*n* = 3 to 6). (F and G) Representative plots depicting reporter expression in splenic donor cells together with bar graphs indicating reporter-positive fractions (*n* = 6). Pooled from three independent experiments. (**H**) The Venn diagrams display TCR clonotypes of T cell subpopulations from three mice. The numbers show those of TCR clonotypes detected in T-bet^−^ MP, T-bet^+^ MP, and/or pT_reg_ cells. (**I** to **K**) T-bet^−^ Rorγt^−^ MP, T-bet^+^ Rorγt^−^ MP, and naïve cells were separately transferred into congenic Foxp3-DTR mice that were subsequently subjected to DT treatment for 10 days. (I) A bar graph showing the number of donor-derived MP cells accumulating in the gut (*n* = 4 to 5). (J and K) Dot plots depicting reporter expression in the indicated donor MP cells in the intestine together with bar graphs displaying the frequency of reporter-positive cells among the donor cells (*n* = 4 to 5). Pooled from three independent experiments. Bar graphs: means ± SD, each symbol represents an individual mouse. *P < 0.05, **P < 0.01, ***P < 0.001.

On the basis of the observation that Foxp3^−^ MP cells comprise undifferentiated as well as T_H_1-differentiated subsets in steady state, we sought to determine the different capacities of T-bet^−^ and T-bet^+^ MP cells to generate T_H_1, T_H_17, and T_reg_ cells under lymphopenic environments. We sorted for the two MP subsets (both Rorγt^−^ Foxp3^−^) from T-bet/Rorγt/Foxp3 triple reporter mice and separately transferred into *Rag2*^−/−^ mice. Four weeks after transfer, the T-bet^−^ MP subset gave rise to higher number of total donor cells especially in the colon (Fig. 7A), and the same lymphocyte population efficiently differentiated into T-bet^+^, Rorγt^+^, and Foxp3^+^ cells, whereas the T-bet^+^ MP subset largely maintained its original phenotype (Fig. 7, B and C). Furthermore, when these two types of donor cells were labeled with CellTrace Violet (CTV), transferred into *Rag2*^−/−^ mice, and analyzed for their CTV dilution 1 week later, T-bet^−^ MP donor cells generated a higher fraction of CTV^−^ cells (fig. S7). Thus, T-bet^−^ as compared to T-bet^+^ MP cells represent a more proliferative precursor with high differentiation potential that can generate T_H_1, T_H_17, and T_reg_ cells in lymphopenic environments. Although T-bet^−^ versus T-bet^+^ MP cells had lower TCR affinity to self-antigens (Fig. 6), inferiority in self-reactivity of the T-bet^−^ MP subset seems to be countered by its higher proliferative potential.

Last, we asked whether T-bet^−^ MP cells can give rise to T_H_1, T_H_17, and T_reg_ cells in more physiological, lymphosufficient environments. In the single-cell RNA sequencing data in Fig. 6A, trajectory analysis suggested spontaneous conversion of undifferentiated MP cells into T_H_1-like or T_reg_-like cells over pseudotime (Fig. 7D and fig. S6B). To formally determine the capacity of T-bet^−^ MP cells to differentiate into T-bet^+^ and other T cell subsets in steady state, we transferred T-bet^−^ Rorγt^−^ MP, T-bet^+^ Rorγt^−^ MP, or naïve cells into congenically marked WT hosts and analyzed donor-derived MP cells 10 days later. Although the number of donor MP cells recovered was very low (Fig. 7E), splenic T-bet^−^ MP cells converted to T-bet^+^, Rorγt^+^, and to a lesser extent Foxp3^+^ cells, while their T-bet^+^ counterparts remained T-bet^+^ Rorγt^−^ Foxp3^−^ (Fig. 7, F and G). Thus, T-bet^−^ Rorγt^−^ Foxp3^−^ MP CD4^+^ T lymphocytes can give rise to T_H_1 and T_H_17 as well as minor T_reg_ cell populations in steady state, although at a less efficient rate. This notion is further supported by the finding that some TCRs were shared by T-bet^−^ MP, T-bet^+^ MP, and/or pT_reg_ cells at homeostasis (Fig. 7H).

Because differentiational as well as proliferative capacities of undifferentiated MP CD4^+^ T cells were to some extent inhibited in lymphosufficient conditions, we hypothesized that preexisting CD4^+^ T lymphocytes inhibit the potential of uncommitted MP cells in these responses. In the case of naïve T lymphocytes, it has been reported that preexisting CD4^+^ T cells can inhibit proliferation of naïve CD4^+^ T cells in *Rag2*^−/−^ mice (*50*). To examine whether preexisting CD4^+^ T cells can suppress MP cell immune responses, we transferred T-bet^−^ or T-bet^+^ MP cells into *Rag2*^−/−^ mice that had received total CD4^+^ T lymphocytes 3 weeks in advance or been left intact. As shown in fig. S8, prior CD4^+^ T cell transfer inhibited expansion of both T-bet^−^ and T-bet^+^ MP cells, suggesting that certain type(s) of preexisting CD4^+^ T lymphocytes can inhibit undifferentiated MP cell immune responses.

We hypothesized that preexisting T_reg_ cells can constrain the potential of undifferentiated MP cells in their proliferation and differentiation. To test this possibility, we transferred T-bet^−^ MP, T-bet^+^ MP, or naïve cells (all Rorγt^−^ Foxp3^−^) into congenically marked Foxp3-DTR mice that were subsequently subjected to DT treatment. Ten days later, T-bet^−^ MP cells expanded more efficiently than T-bet^+^ MP cells in the environment where preexisting T_reg_ cells were depleted (Fig. 7I). Furthermore, T-bet^−^ MP cells efficiently converted to T-bet^+^, Rorγt^+^, and Foxp3^+^ cells (Fig. 7, J and K). Therefore, undifferentiated MP CD4^+^ T lymphocytes exert their potential to proliferate and differentiate into T_H_1, T_H_17, and T_reg_ cells especially in the absence of T_reg_ cells.

## DISCUSSION

In the present study, we have defined the behaviors of MP CD4^+^ T lymphocyte subsets in lymphopenic, lymphosufficient, and T_reg_-depleted inflammatory environments. Our data demonstrate that self-reactive MP cells as a whole population have a potential to expand in multiple organs including colon and lungs in lymphopenic conditions, and, particularly in the intestine, the same lymphocytes differentiate into T_H_1 and T_H_17 cells in an IL-12/23–dependent manner while responding to TGFβ to differentiate into T_reg_ cells thereby contributing to pathogenesis of inflammation. Moreover, MP cells comprise not only T_H_1/17-differentiated but also T-bet^−^ Rorγt^−^ subsets in steady state, with the latter representing a polyclonal, transcriptomically undifferentiated subpopulation. Furthermore, while the T-bet^+^ MP subset is already terminally differentiated into T_H_1 and shows no plasticity to T_H_17/T_reg_ cells, T-bet^−^ cells have a potential to efficiently proliferate and differentiate into T_H_1, T_H_17, and T_reg_ cells, the response of which is constrained by preexisting T_reg_ cells in steady state. Together, our results reveal previously unappreciated heterogeneity of MP CD4^+^ T lymphocytes and identify the undifferentiated MP subset as a precursor with a diverse differentiation potential that can give rise to T_H_1, T_H_17, and T_reg_ cells to mediate mild and persistent inflammation.

It is well known that homeostasis of naïve and MP CD4^+^ T lymphocytes is maintained by a proliferative response referred to as “homeostatic proliferation” in the periphery (*1*, *2*). Thus, once generated in the thymus, peripheral naïve CD4^+^ T cells exhibit two types of proliferation: slow and fast cell divisions (*51*, *52*). While slowly proliferating cells largely maintain their CD44^lo^ phenotype, the fast-dividing cell population spontaneously converts to MP cells, the response of which is best examined in an artificial, lymphopenic environment (*51*–*54*) but can also occur in physiological, lymphosufficient conditions (*6*, *55*). The fast homeostatic proliferation requires antigen recognition as well as costimulatory signals and, indeed, is significantly diminished in the absence of TCR, CD28, or OX40 signaling (*6*, *53*, *56*–*60*). Under lymphopenic environments, the expansion is dampened when commensal microflora are reduced or absent (*52*, *56*, *61*), demonstrating that commensal antigens play an essential role in the response generation. Consistent with this notion, naïve CD4^+^ T lymphocytes can induce severe colitis dependently of commensal microbiota present in chronically lymphodeficient hosts (*25*–*27*, *30*), arguing that excess lymphopenia-induced proliferation of naïve cells can lead to immunopathology.

In contrast to naïve cells, there is dearth of information regarding homeostatic proliferation of MP CD4^+^ T lymphocytes. It is known that when transferred into lymphopenic mice, MP cells themselves exhibit slow and fast cell divisions (*57*, *62*, *63*). The former response is dependent on IL-7, whereas the latter requires Ag recognition as well as OX40 signaling. Furthermore, we previously reported that while slowly proliferating cells exhibit type 1 differentiation, the fast-dividing cell population can give rise to T_H_17 cells (*62*). However, it was unclear whether these proliferative responses can trigger inflammation in vivo.

Our present study shows that MP CD4^+^ T cells expand in multiple organs including the colon to generate inflammatory responses in lymphopenic environments, suggesting a pathological aspect of MP cell homeostatic proliferation. Similarly to naïve CD4^+^ T cell–induced colitis (*31*, *32*), we found that MP cells contribute to pathogenesis of colitis in a manner dependent on type 1 and 3 immune responses. Thus, while WT, *Ifng*^−/−^, and *Il17a*^−/−^ MP cells exhibited unaltered degree of inflammation, deficiency in IFN-γ in the same cells significantly inhibited colitis when IL-17A was blocked (Fig. 2), arguing that MP cell–derived IFN-γ plays an important role in the response generation. A likely explanation is that IFN-γ and IL-17A produced by MP cells act as effector cytokines in a cooperative or compensatory manner in the colon given that some MP cells produced both IFN-γ and IL-17A in lymphopenic settings and that production of the latter cytokine was augmented in the absence of the former. The other possibility is that IFN-γ produced by MP cells augments intestinal inflammation in the influence of non–T cell–derived IL-17A. In the case of naïve cell–induced colitis, IL-17A of T cell as well as type 3 ILC origins redundantly contribute to pathogenesis of inflammation (*64*), suggesting that ILC3s also play a role in the MP cell responses through IL-17A production in the gut. In addition, our present data suggest that IFN-γ of non–T cell origins could also contribute to MP cell immune responses because anti–IFN-γ mAb significantly reduced MP cell accumulation in the spleen (fig. S5A), whereas *Ifng*^−/−^ and WT MP cells equally infiltrated into the same organ (Fig. 2B). Thus, IFN-γ derived from NK cells or ILC1s may further augment the MP cell–mediated immune responses.

In contrast to intestinal inflammation, molecular mechanisms of MP cell–driven lung inflammation are unclear. Because MP cells induced unaltered degree of lung inflammation in the absence of IFN-γ and IL-17A (Fig. 2), other cytokines may play a role in the response generation. In this regard, lung tissues are known to be abundant in type 2 cytokines (*65*), whereas T_H_1 and T_H_17 responses are dominant in the gut (*31*). It is therefore possible that MP cells and especially their undifferentiated subset respond to TSLP/IL-33/25 and induce inflammatory responses in an IL-4/5/13–dependent manner in the lung.

To which antigens, self or foreign, MP cells respond is an important question. In lungs and spleen, accumulation of self-reactive MP CD4^+^ T lymphocytes was not diminished in GF conditions (Fig. 1), suggesting that MP cells may, by default, use available antigens including self for their response generation in relatively sterile environments (*66*). By contrast, in the colon, SPF MP cells did not induce colitis in GF *Rag1^−/−^* mice, whereas GF MP cells did develop colitis in SPF *Rag1^−/−^* animals. Given that the total cell number and TCR repertoire diversity of splenic MP cells in steady state were not affected by the presence of commensal microflora (Fig. 1L) (*4*, *5*), the following two mutually nonexclusive possibilities are likely. First, MP cells originally generated from naïve precursors through self-antigen recognition cross-react with foreign antigens that are abundantly present in the colon. Given a positive correlation between TCR affinity to self-antigens and that to foreign antigens in T lymphocytes (*41*, *42*), MP cells that can otherwise only weakly respond to self may strongly react with foreign antigens in the gut environment. The other possibility is that MP cells more strongly respond to self-antigens in the presence of commensal microbiota–derived components with adjuvant-like activity that exist in abundance in the colon. Various types of commensal-derived agonists including lipopolysaccharide have been shown to promote maturation of dendritic cells by enhancing their antigen processing and presentation as well as expression of costimulatory molecules (*67*, *68*). In such circumstances, self-directed responses of MP cells may be heightened.

In this study, we revealed that in addition to the terminally T_H_1/17-differentiated subsets we and other groups previously reported (*6*, *13*, *14*), MP CD4^+^ T lymphocytes contain a T-bet^−^ Rorγt^−^ Foxp3^−^ undifferentiated subset in steady state and that this subpopulation has a potential to give rise to not only T_H_1/17 effectors but also T_reg_ cells. Given inflammatogenic nature of MP cells, this machinery appears to serve as a safeguard to prevent autoimmune and inflammatory diseases. This poses an intriguing question of when and how the fate of MP CD4^+^ T lymphocytes to differentiate into T_reg_ versus T_H_1/17 cells is determined. In the process of MP cell development in steady state, upon self-antigen recognition, naïve precursors robustly proliferate to acquire a CD44^hi^ CD62L^lo^ phenotype, followed by acquisition of high T-bet expression as well as quiescent states (*5*, *6*, *13*). Consistent with this, our present study shows that T-bet^−^ Rorγt^−^ MP cells can be directly generated from naïve precursors and exhibit more rapid proliferation than do their T-bet^+^ Rorγt^−^ counterparts in steady state (Fig. 6). Furthermore, while T-bet^−^ Rorγt^−^ MP cells retain the capacity to differentiate into T_H_1/17 effector and T_reg_ cells, T-bet^+^ Rorγt^−^ MP lymphocytes have been terminally T_H_1-differentiated (Fig. 7). These observations argue that while rapidly proliferating, undifferentiated MP cells have both inflammatory and immunosuppressive potentials, they lose their capacity to convert to T_reg_ cells as they differentiate into more quiescent T_H_1 cells. It remains to be determined whether undifferentiated MP cells are actively maintained through proliferative responses once generated at homeostasis, or whether these cells generated from naïve precursors eventually all differentiate into the T_H_1/17-like subsets.

Do all T-bet^−^ Rorγt^−^ MP cells belong to the undifferentiated subset? Our present data suggest the existence of “multipotent” cells within this MP subpopulation because T-bet^−^ Rorγt^−^ but not T-bet^+^ Rorγt^−^ MP cells gave rise to substantial fractions of T_H_1, T_H_17, and T_reg_ cells and because some TCRs were shared by T-bet^−^ Rorγt^−^ MP, T-bet^+^ Rorγt^−^ MP, and pT_reg_ cells at homeostasis (Fig. 7). Although the population size of such TCR clones was small in steady state, multipotency of undifferentiated MP cells may be inhibited in the presence of MP-extrinsic T_reg_ cells. Nevertheless, these observations do not necessarily mean that all T-bet^−^ Rorγt^−^ MP cells present at homeostasis belong to a pure, undifferentiated population. Because a small fraction of T-bet^+^ MP cells down-regulated their T-bet expression in steady state (Fig. 7F), T-bet^−^ Rorγt^−^ MP cells may contain a subpopulation that had previously expressed T-bet but shut down its expression. In this regard, in the case of conventional, antigen-specific responses, a substantial fraction of “ex–T-bet” cells have been reported to adopt a T follicular helper cell (T_FH_) phenotype (*69*). It may thus be possible that such T_FH_-like cells exist in the T-bet^−^ Rorγt^−^ MP cell population and have unique immunological function. Further investigation will be necessary to elucidate heterogeneity of T-bet^−^ Rorγt^−^ MP cells.

Is the fate of thymocytes to eventually differentiate into MP versus T_reg_ cells predetermined at a thymic positive selection stage? This seems to be the case because thymocytes with unique TCRs that can receive relatively strong signals from self-MHC ligands differentiate into tT_reg_ cells (*29*) and because among the rest of the self-reactive clones, only those that have relatively high TCR affinity to self-antigens can generate MP cells and especially their T-bet^+^ subset in the periphery, the response of which has been confirmed at both polyclonal and monoclonal levels (*6*, *13*). This notion is further supported by our present data showing that different combinations of hydrophobic amino acid residues that potentially promote self-reactivity of T cells (*48*, *49*) are enriched in the TCRβ CDR3 regions of tT_reg_ versus T-bet^+^ MP cells (Fig. 6). However, in the present study, we found that T-bet^−^ MP cells have a substantially diverse TCR repertoire. It is therefore possible that naïve cells that have found their cognate self-antigens randomly presented on dendritic cells (*70*) proliferate to convert to immature MP cells in a stochastic manner as well.

Our findings reported here define naturally arising, undifferentiated MP CD4^+^ T lymphocytes as a polyclonal and proliferative cell population that has both inflammatogenic and immunosuppressive potentials in vivo. On the basis of the observations that undifferentiated MP cells have the capacity to induce T_H_1/17/T_reg_ responses in lymphopenic as well as T_reg_-depleted inflammatory conditions and that this potential is to some extent inhibited in physiologic, lymphosufficient settings, we speculate that the same cells could contribute to the development of certain types of autoimmune and inflammatory diseases, especially in the environment where T_reg_-mediated immunosuppression is weakened. In examining this hypothesis, it is necessary to test immunological functions of undifferentiated MP cells in various autoimmune/inflammatory disease contexts, which needs further investigation in the future.

Last, it is important to determine whether self-driven MP cells are present in humans. Previous reports identify CD4^+^ T lymphocyte populations with an effector/memory phenotype in cord blood and lymphoid as well as extralymphoid organs of the human fetus where an encounter with foreign antigens is likely to be very limited or nonexistent (*71*–*74*). Moreover, rapidly proliferating MP CD4^+^ and CD8^+^ T lymphocyte population with a broad TCR repertoire has been reported to develop even in healthy adults, which further grows up in size in HIV-infected settings (*75*, *76*). Such rapidly dividing MP CD4^+^ T cells have a T_reg_-associated gene expression signature (*77*), strongly suggesting the existence of undifferentiated MP cells in humans. If these human MP cells are functional in inflammatory/immunosuppressive responses, their differentiation and activation pathways could be targeted as a therapeutic strategy for treatment of autoimmune and inflammatory diseases.

## MATERIALS AND METHODS

### Study design

The aim of this study was to characterize previously unappreciated heterogeneity of MP CD4^+^ T lymphocytes and analyze inflammatogenic as well as immunosuppressive functions of each MP subset. For this purpose, in vivo and in vitro experiments using mice were performed as described here in Materials and Methods. The animal experiments were not randomized. The investigators were not blinded to the allocation during experiments and analyses. Experimental replication is indicated in the figure legends.

### Mice

C57BL/6 WT mice were purchased from Japan SLC (Hamamatsu, Japan) or Taconic Biosciences (Rensselear, NY). *Rag2^−/−^*, *Rag1^−/−^*, and CD45.1 WT mice were obtained from breeding stocks at Tohoku University Graduate School of Medicine, Pohang University of Science and Technology (POSTECH), or the National Institute of Allergy and Infectious Diseases (NIAID) contract facility at Taconic Biosciences. Foxp3-RFP reporter mice (*78*) were purchased from the Jackson Laboratory (Bar Harbor, ME). T-bet-ZsGreen/Rorγt-E2Crimson/Foxp3-RFP triple reporter mice were generated by breeding Foxp3-RFP mice with the T-bet-ZsGreen/Rorγt-E2Crimson strain (*79*, *80*) and obtained from the NIAID contract facility at Taconic Biosciences. *Ifng*^−/−^ and *Il17a*^−/−^ mice are previously described (*81*, *82*). Foxp3-DTR-4 mice (*83*) were provided by G. J. Hammerling (German Cancer Research Center, Heidelberg, Germany). All mice were maintained in SPF animal facilities in Tohoku University Graduate School of Medicine or NIAID except for GF mice, which were bred and maintained in the Microbiome Core Facility of POSTECH as previously described (*4*). All mice were used at the age of 8 to 20 weeks. The care and handling of the animals used in our studies were in accordance with the animal study protocols approved by the Institutional Committee for the Use and Care of Laboratory Animals of Tohoku University (2019-127-06, 2019-128-05, 2019-129-06, 2019-260-08), by the Institutional Animal Care and Use Committee of POSTECH (POSTECH-2020-0069), or by the NIAID Animal Care and Use Committee (LISB-8E).

### Cell sorting and adoptive transfer

Total CD4^+^ T lymphocytes were obtained from pooled splenocytes of donor mice using CD4 Microbeads (Miltenyi Biotec, Bergisch Gladbach, Germany). MP cells were then purified by sorting for CD4^+^ Foxp3-RFP^−^ CD44^hi^ CD62L^lo^ or, in some cases (Fig. 2, B to F, fig. S4, A to D), CD4^+^ CD25^−^ CD44^hi^ CD62L^lo^ population using FACS Aria II (BD Biosciences, San Jose, CA). In Figs. 6 and 7, T-bet-ZsGreen^−^ Rorγt-E2Crimson^−^ and T-bet-ZsGreen^+^ Rorγt-E2Crimson^−^ cells were further purified. To obtain naïve and T_reg_ cells, CD4^+^ Foxp3-RFP^−^ CD44^lo^ CD62L^hi^ and CD4^+^ Foxp3-RFP^+^ populations, respectively, were sorted out. Purity was >95% (gating strategies are shown in fig. S9, A and B). In fig. S7, donor cells were labeled with CTV (Thermo Fisher Scientific, Waltham, MA) following the manufacturer’s protocol. For adoptive transfer, 5 × 10^5^ cells were intravenously injected into sex-matched recipient mice.

### In vivo mAb and chemical treatments

To block IFN-γ, IL-12/23 p40, or TGFβ, anti–IFN-γ (XMG1.2), anti–IL-12/23 p40 (C17.8), anti-TGFβ (1D11.16.8) (all from Bio X Cell, West Lebanon, NH), or control immunoglobulin G (Sigma-Aldrich, St. Louis, MO) were intraperitoneally administered every 3 days (300 μg/20 g body weight). To inhibit IL-17A, anti–IL-17A mAb (TC11-18H10.1) (BioLegend, San Diego, CA) was intraperitoneally injected every other day (50 μg/20 g body weight). To deplete donor-derived T_reg_ cells, *Rag2^−/−^* animals that had been transferred with MP/naïve cells from Foxp3-DTR or control Foxp3-RFP mice received DT intraperitoneally every other day (0.5 μg/20 g body weight).

### Clinical examination of colitis and lung inflammation

The disease activity index score was assessed on the basis of clinical symptoms as previously described (*84*). Blood arterial oxygen saturation was measured using a mouse pulse oximeter sensor (MouseOx Plus, Starr Life Sciences Corp, Allison Park, PA) according to the manufacturer’s instructions.

### Serum biochemical analysis

Whole blood was obtained from *Rag2*^−/−^ recipient mice and subjected to serum biochemical analysis. The analysis was performed by Oriental Yeast (Tokyo, Japan).

### Histological assessment of tissue damage

Approximately four weeks after donor cell transfer into *Rag2^−/−^* mice, histological assessment of colon, lungs, kidneys, and liver was performed by H&E and immunohistochemical staining. For the latter staining, rabbit CD4 mAb (EPR19514; diluted in 1:2000) (Abcam, Cambridge, UK) and secondary anti-rabbit antibody conjugated with peroxidase (Nichirei Biosciences, Tokyo, Japan) were used to visualize CD4^+^ cells.

Severity of histological colitis was evaluated using mouse colitis histology index as previously reported (*85*). Briefly, the following four histological components were assessed: goblet cell loss (0 = none, 1 = <10%, 2 = 10 to 50%, and 3 = >50%), crypt density (0 = normal, 1 = decrease of <10%, and 2 = decrease of >10%), crypt hyperplasia (0 = none, 1 = slightly increased crypt length, 2 = 2 to 3 times increased crypt length, and 3 = >3 times increased crypt length), and submucosal infiltration (0 = none, 1 = individual cells, 2 = infiltrates, and 3 = large infiltrates). Mouse colitis histology index was calculated as 1 × goblet cell loss + 2 × crypt density + 2 × crypt hyperplasia + 3 × submucosal infiltration. For histological assessment of interstitial pneumonia in a perivascular region, five blood vessels in lung tissues were randomly chosen and scored according to the following criteria; 0: no donor cells seen in a perivascular region; 1: a few donor cells seen in a perivascular region; 2: one to two layer(s) of donor cells surrounding the vessel; 3: three to four layers of donor cells surrounding the vessel; 4: five to six layers of donor cells surrounding the vessel; 5: seven or more layers of donor cells surrounding the vessel. Lung inflammation histology index was defined as a sum of the scores at the five vessels.

### Flow cytometric analysis

Single-cell suspensions were prepared from spleens and red blood cells lysed in ACK buffer. To obtain colonic lamina propria cells, Lamina Propria Dissociation Kit (Miltenyi Biotec) was used following the manufacturer’s protocol. To isolate lung cells, lungs were perfused with 20 ml of phosphate-buffered saline (PBS), followed by tissue digestion with Multi Tissue Dissociation Kit 1 (Miltenyi Biotec) following the manufacturer’s instruction. In fig. S2, mice received intravenous injection of CD4-FITC mAb (RM4-4) 5 min before euthanasia and were subjected to lung tissue digestion without perfusion.

For intracellular cytokine detection, lymphocytes were incubated in RPMI 1640 complete media containing phorbol 12-myristate 13-acetate (20 ng/ml) and ionomycin (1 μg/ml) (both from Fujifilm Wako, Osaka, Japan) for 4 hours in the presence of brefeldin A (BioLegend) following procedures we previously described (*86*). The cells were then suspended in PBS supplemented with 2% fetal bovine serum and incubated with CD16/32 mAb for 10 min on ice, followed by incubation with the following mAbs or their combination for 20 min on ice: CD3 (17A2), CD4 (RM4-5), CD25 (PC61), CD44 (IM7), CD45RB (16A), CD62L (MEL-14), (BD Biosciences), CCR7 (4B12), CD5 (53-7.3), CD27 (LG.3A10), NK1.1 (PK136), and Nrp1 (3E12) (BioLegend). To detect intracellular products, the cells were further fixed and permeabilized using Foxp3/Transcription Factor Staining Buffer Set for 20 min on ice and stained with mAbs against Foxp3 (FJK-16s), Ki67 (SolA15) (Thermo Fisher Scientific), IFN-γ (XMG1.2), IL-17A (TC11-18H10.1), and/or TCF1 (S33-966) (BD Biosciences) for 20 min on ice. Flow cytometry was performed using LSR Fortessa and the data analyzed with FlowJo software (both BD Biosciences). Gating strategies are detailed in fig. S9.

### Reanalysis of single-cell RNA sequencing data

We previously performed single-cell RNA sequencing of TCRβ^+^ CD4^+^ CD25^−^ CD44^hi^ CD62L^lo^ cells and deposited the dataset on Gene Expression Omnibus (GEO) under the accession number GSE145999 (*5*). In brief, the sorted cells were encapsulated into droplets, and libraries were prepared using Chromium Single Cell 3′ Reagent Kits v2 according to the manufacturer’s protocol (10X Genomics). The generated libraries were sequenced on the Illumina NextSeq using paired-end 26 × 98 base pairs, and sequencing files were processed to extract count matrices using the Cell Ranger Single-Cell Software Suite (v2.2.0). In the present study, we reanalyzed the data with R (v4.2.2) using the Seurat package (v4.3.0) (*87*). Specifically, we log-normalized the expression matrix, regressed the data against the total number of unique molecular identifiers detected per cell, and performed PCA analysis to find clusters. Minor CD3e^+^ NK1.1^+^ NKT cell cluster was removed for downstream analyses. We visualized single-cell gene expression as UMAP overlays, signature scores, and dot plots. T_H_1, T_H_17, T_reg_, and ISG^hi^ gene signatures were defined as shown in table S1, and the scores calculated by Seurat using “AddModuleScore” function. Single-cell trajectory was analyzed by Monocle 3 (*88*).

### TCR repertoire analysis

T-bet^+^ and T-bet^−^ MP (CD3^+^ CD4^+^ Foxp3-RFP^−^ CD44^hi^ CD62L^lo^ T-bet-ZsGreen^+^ Rorγt-E2Crimson^−^ and CD3^+^ CD4^+^ Foxp3-RFP^−^ CD44^hi^ CD62L^lo^ T-bet-ZsGreen^−^ Rorγt-E2Crimson^−^, respectively) and naïve (CD3^+^ CD4^+^ Foxp3-RFP^−^ CD44^lo^ CD62L^hi^) CD4^+^ T lymphocytes as well as pT_reg_ and tT_reg_ cells (CD3^+^ CD4^+^ Foxp3-RFP^+^ Nrp1^−^ and CD3^+^ CD4^+^ Foxp3-RFP^+^ Nrp1^+^, respectively) were obtained from spleens of T-bet/Rorγt/Foxp3 triple reporter mice. For the repertoire analysis of MP cells transferred into *Rag2*^−/−^ mice, Foxp3-RFP^−^ and Foxp3-RFP^+^ donor cells were recovered from the colon 4 weeks after transfer. Genomic DNA was purified from the sorted cells using DNeasy Blood & Tissue Kit (QIAGEN, Hilden, Germany). TCRβ CDR3 regions were amplified and sequenced by Adaptive Biotechnologies (Seattle, WA).

To calculate the normalized Shannon index, we divided the Shannon index by its theoretical maximum, log(*k*), where *k* is the number of clonotypes, using R (v4.2.2). The normalized index takes a value between 0 and 1 and is comparable across datasets with different numbers of clonotypes. For quantification of the frequency of clonally expanding clones, we defined clonally expanding clones as those that occupy more than 0.1% of the total sequencing reads and quantified the fraction of reads originating from them in each dataset. To generate Venn diagrams, R with Phyloseq and Venneuler was used.

For Vβ gene as well as CDR3 amino acid analyses (Fig. 6), we also downloaded a publicly available dataset from the Adaptive Biotechnologies website (https://adaptivebiotech.com/) (*89*) and included “Helios+T_reg_” (tT_reg_), “Helios-T_reg_” (pT_reg_), and “Tnaive” (naïve CD4^+^ T cells) populations in the data obtained above to capture robust TCR signature of each T cell subpopulation. In these analyses, we used unique clonotypes in each sample to exclude the effect of clonal expansion. For Vβ gene analysis, we calculated the usage ratio for each gene in each sample. CDR3 amino acid analysis was conducted following the same strategy as described in our previous study (*48*). Briefly, this strategy focuses on middle amino acid positions (P108 to P112), jointly analyzing these positions across all lengths of CDR3. We obtained one average frequency for each amino acid in each sample. The code to calculate amino acid frequencies is available on our GitHub (https://github.com/immunogenomics/cdr3-QTL). We conducted PCA analysis using “prcomp” function in R.

### Statistical analysis

A two-sided Student’s *t* test was performed to establish statistical significance between the two groups. For multiple comparisons, one-way analysis of variance (ANOVA) followed by Dunnett’s or Tukey’s tests were used. To assess correlation between two variables, Spearman’s rank correlation coefficient (ρ) was calculated. *P* values <0.05 were considered significant.
