# Supplementary material for: Naturally arising memory-phenotype CD4+ T lymphocytes contain an undifferentiated population that can generate TH1, TH17, and Treg cells
Source: Sci Adv. 2024 Dec 4;10(49):eadq6618. doi: 10.1126/sciadv.adq6618 (PMC11619248; doi:10.1126/sciadv.adq6618)
Supplement: Supplementary file 1 — Figs. S1 to S9 Table S1 Legend for data S1 [file sciadv.adq6618_sm.pdf]

Science Advances
AAAS

Supplementary Materials for

Naturally arising memory-phenotype $CD4^+$ T lymphocytes contain an undifferentiated population that can generate T_H1, T_H17, and Treg cells

Akihisa Kawajiri *et al.*

Corresponding author: Takeshi Kawabe, kawabet@tohoku.ac.jp

Sci. Adv. **10**, eadq6618 (2024)
DOI: 10.1126/sciadv.adq6618

The PDF file includes:

Figs. S1 to S9
Table S1
Legend for data S1

Other Supplementary Material for this manuscript includes the following:

Data S1

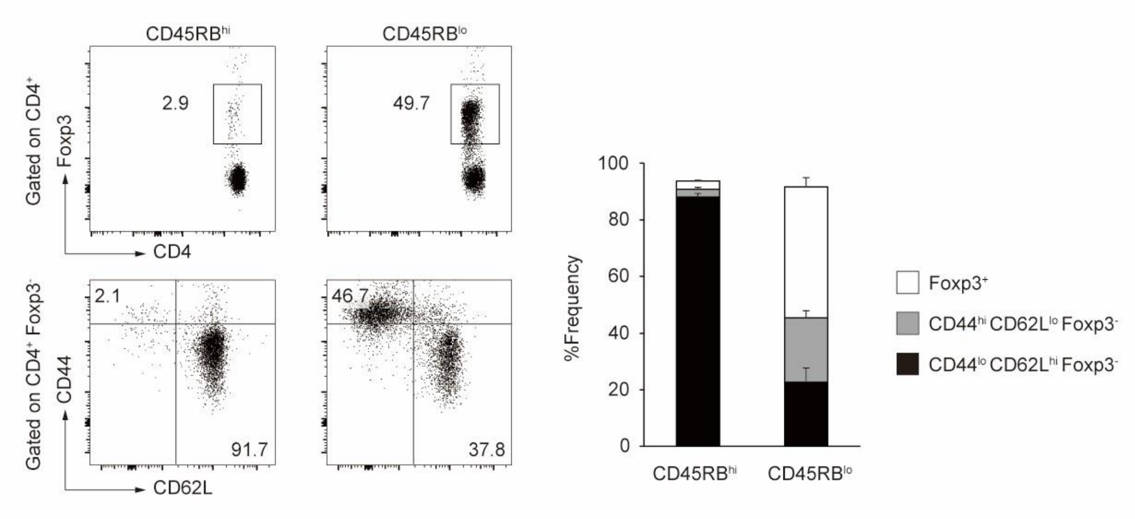

Fig. S1. CD45RB^{lo} CD4^{+} T lymphocytes comprise naïve, MP, and Treg cells whereas their CD45RB^{hi} counterparts are essentially all naïve.

Representative dot plots display expression of CD44, CD62L, and Foxp3 in CD45RB^{hi} versus CD45RB^{lo} CD4^{+} T lymphocytes whereas the bar graph shows the frequency of CD44^{lo} CD62L^{hi} Foxp3^{-} naïve, CD44^{hi} CD62L^{lo} Foxp3^{-} MP, and Foxp3^{+} Treg subpopulations among the indicated CD4^{+} T cell subpopulations (n=3 mice). Representative of two independent experiments. Bar graph: mean ± S.D.

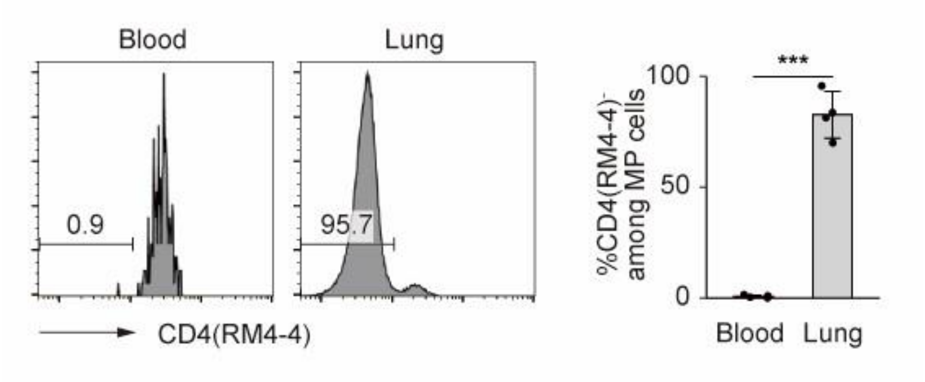

Fig. S2. The vast majority of MP donor cells detected in the lung of *Rag2*$^{-/-}$ mice are tissue-resident.

Rag2$^{-/-}$ mice that had received MP cells 4 weeks in advance were subjected to the iv staining with CD4 (RM4-4) mAb and analyzed for its level on donor cells in the blood and lung (detailed in Materials and Methods). The representative histograms display CD4 (RM4-4) levels on MP cells whereas the bar graph indicates the fraction of CD4 (RM4-4)$^{-}$ cells among the total MP population in the indicated tissues (n=4). Representative of two independent experiments. Bar graph: mean ± S.D, each symbol represents an individual mouse. *** p<0.001.

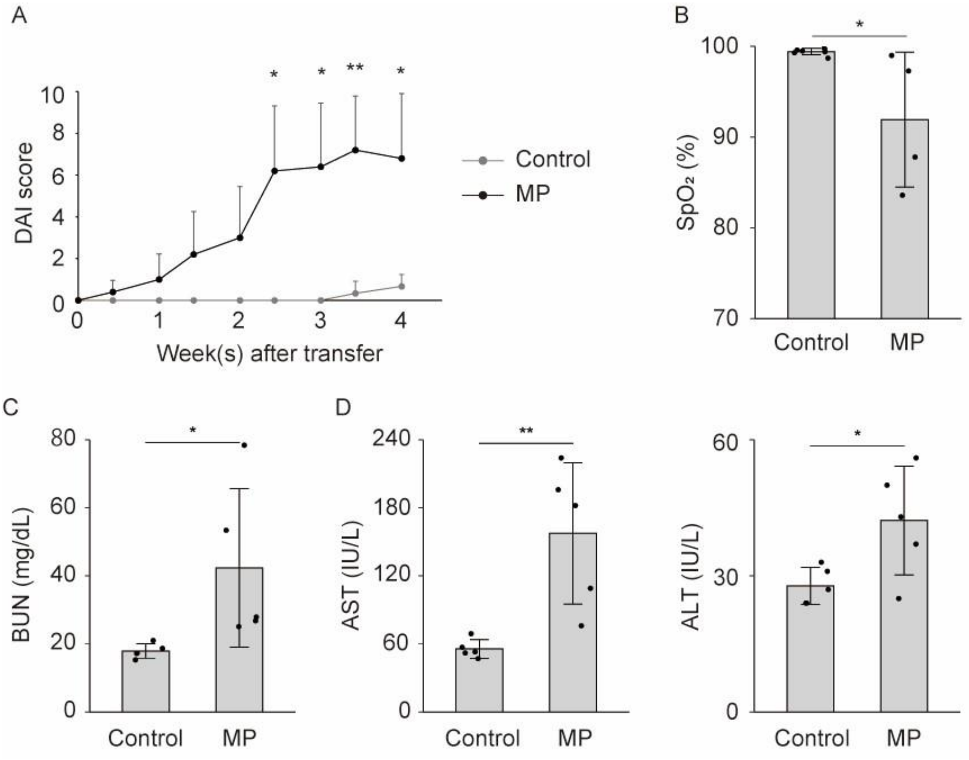

Fig. S3. Clinical behavior and laboratory findings of multi-organ inflammation in *Rag2*$^{-/-}$ mice that have received MP cells.

(**A**) *Rag2*$^{-/-}$ mice that had received MP cells were examined for clinical symptoms of colitis over the course of the experiments. The graph shows the DAI score measured at different time points (n=3-5). (**B**) In the above experiments animals were measured for percutaneous oxygen saturation (SpO_2) (n=4-7). (**C, D**) Bar graphs showing serum concentration of (C) BUN, (D) AST, and ALT four weeks after MP cell transfer (n=5). Pooled from two independent experiments. Bar graphs: mean ± S.D, each symbol represents an individual mouse. * $p<0.05$, ** $p<0.01$.

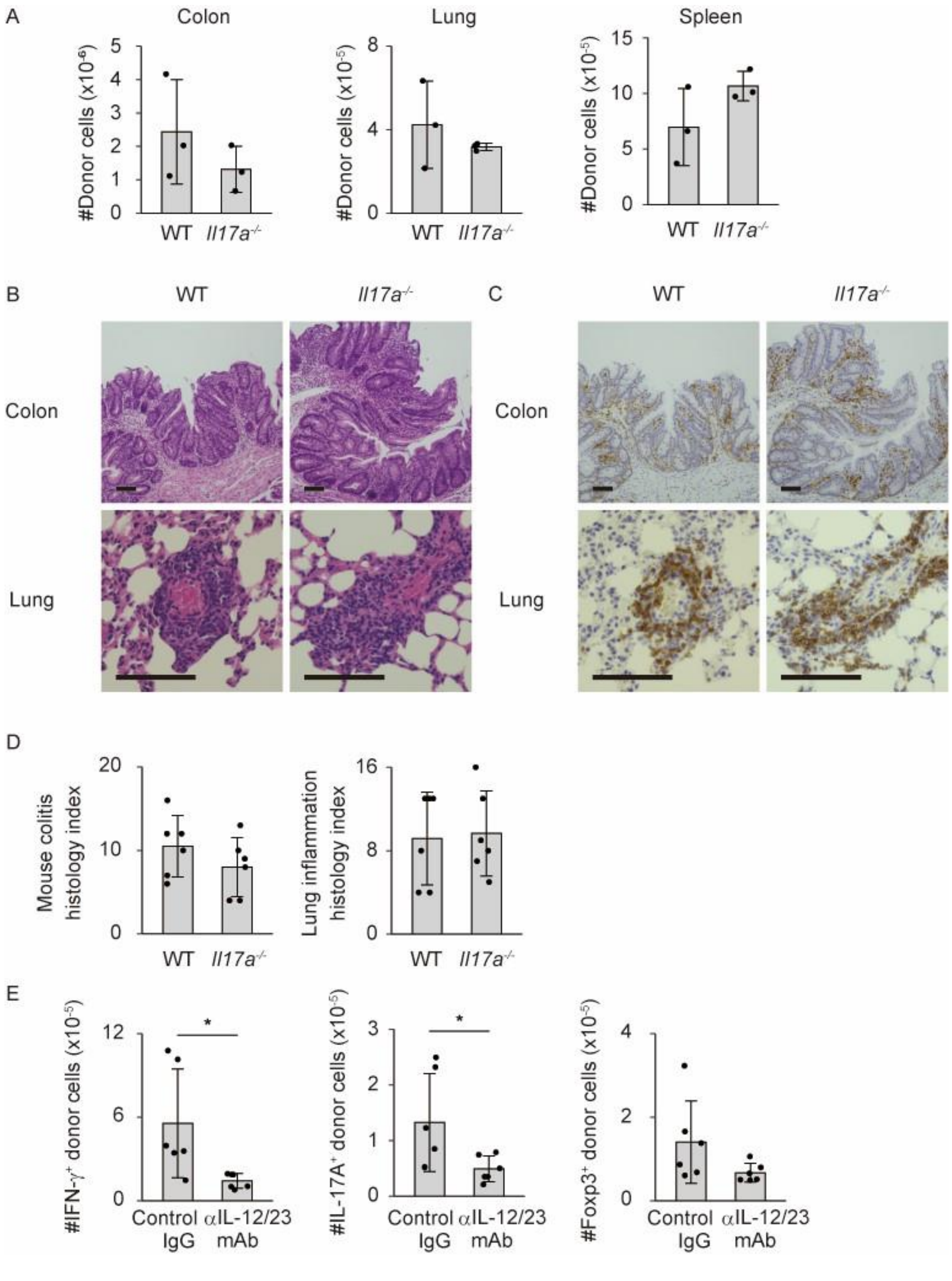

Fig. S4. MP cells accumulate in the intestine and lungs of *Rag2*$^{-/-}$ mice in the absence of MP cell-derived IL-17A.

(**A – D**) WT or *Il17a*$^{-/-}$ MP cells were transferred into *Rag2*$^{-/-}$ mice and the donor cells as well as recipient animals analyzed four weeks later. (A) The bar graphs show the number

of donor cells in the indicated organs (n=3). Representative of two independent experiments. (B – D) Representative images of H&E and CD4-directed immunohistochemical staining as well as bar graphs indicating histological scores are shown (n=6). Pooled from two independent experiments. (**E**) MP cells had been transferred into *Rag2*$^{-/-}$ mice that were subsequently treated with anti-IL-12/23 p40 mAb or control IgG. The bar graphs show the number of IFN-γ^{+}, IL-17A^{+}, and Foxp3^{+} donor cells accumulating in the gut (n=6). Pooled from two independent experiments. Bar graphs: mean ± S.D, each symbol represents an individual mouse. Scale bar: 100 μm. * $p<0.05$.

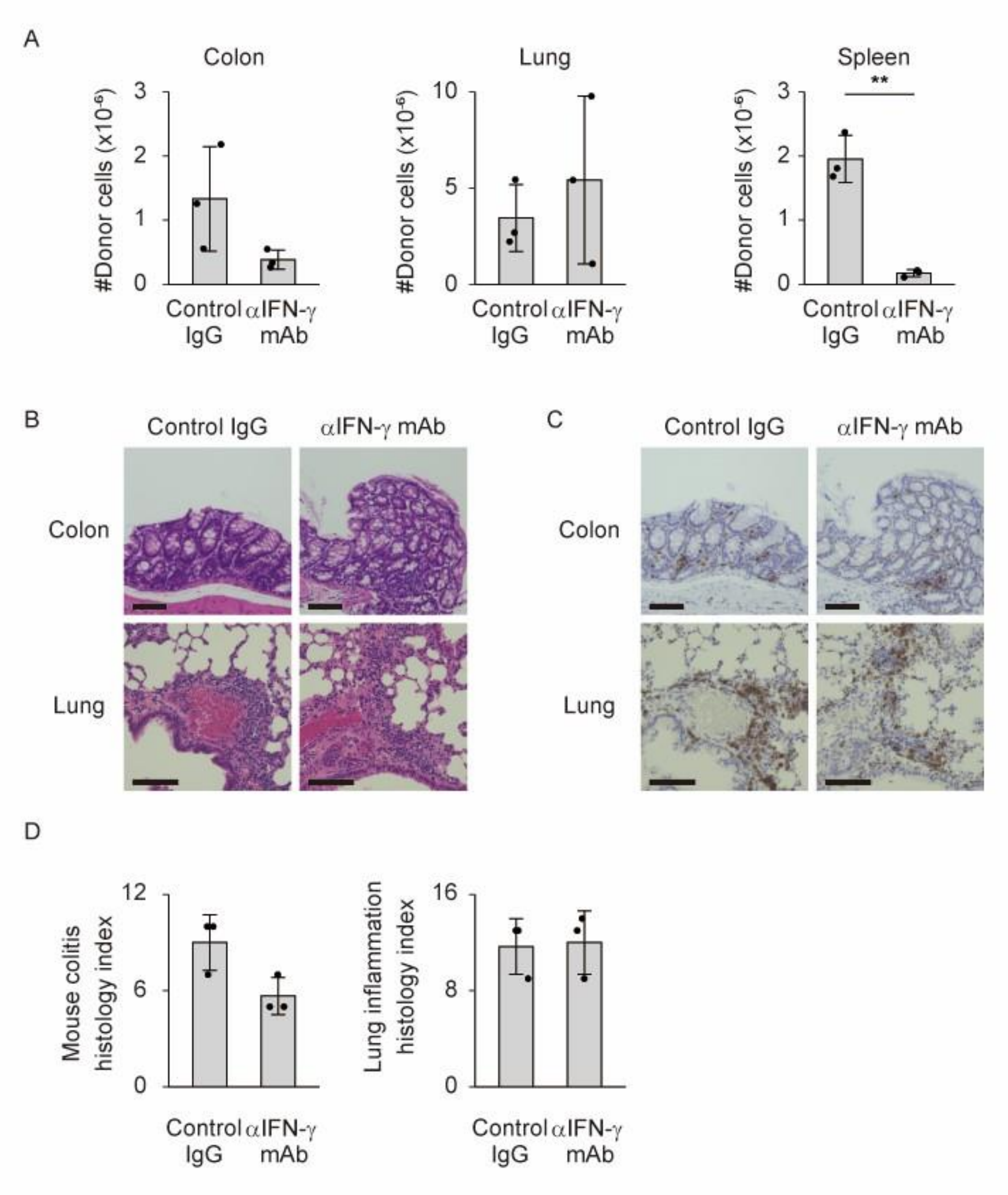

Fig. S5. MP cells can trigger inflammatory responses in the colon and lungs of *Rag2*$^{-/-}$ mice treated with anti-IFN-γ mAb.

(**A**) MP cells were transferred to *Rag2*$^{-/-}$ mice that subsequently received either anti-IFN-γ mAb or Control IgG. The bar graphs show the number of donor cells accumulating in the indicated organs 4 weeks after transfer (n=3). (**B – D**) Representative images of H&E and CD4-directed immunohistochemical staining as

well as bar graphs indicating histological scores of the inflammation are shown (n=3). Bar graphs: mean ± S.D, each symbol represents an individual mouse. Scale bar: 100 μm. ** $p<0.01$.

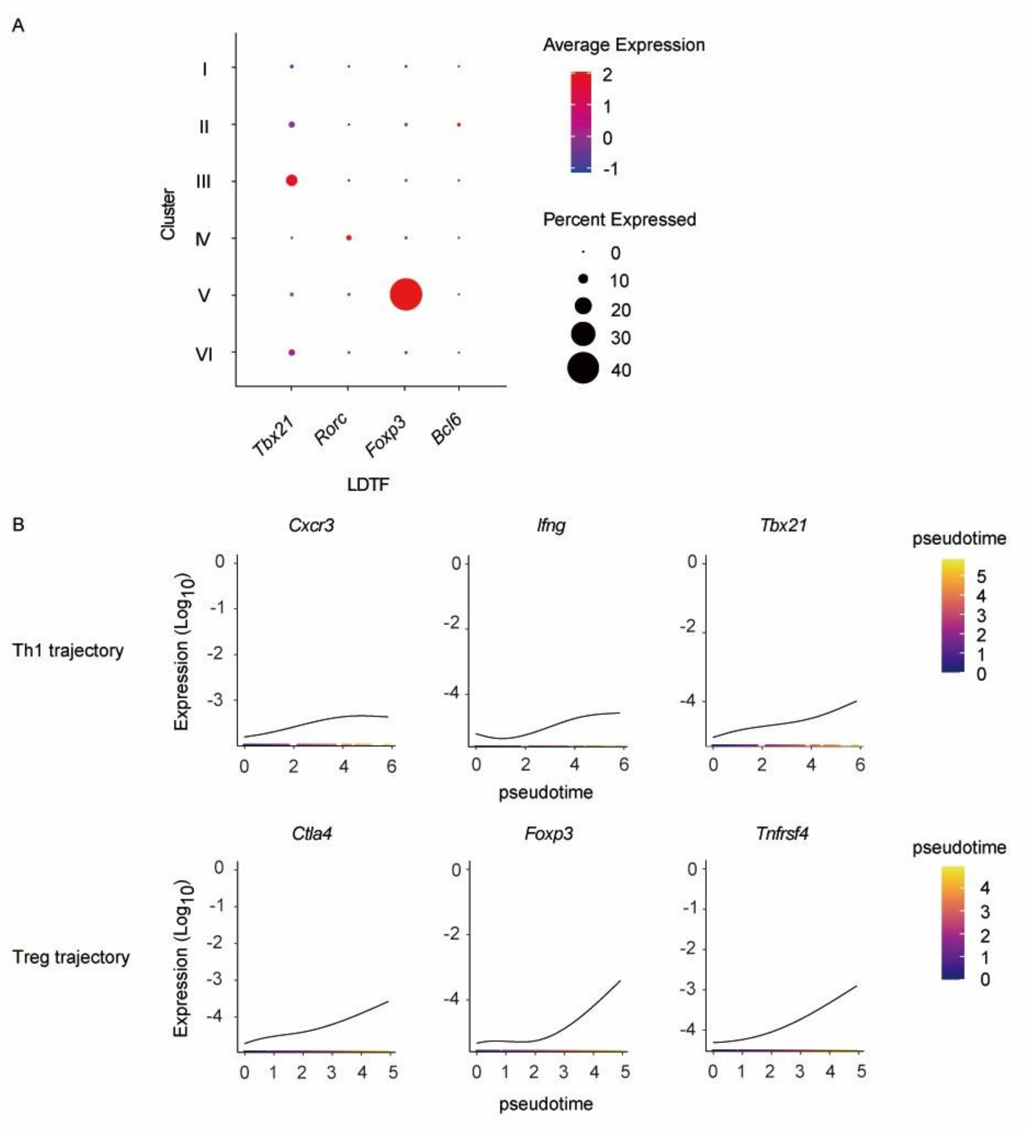

Fig. S6. Some MP clusters express LDTFs.

(**A**) A dot displays both the relative expression level of each LDTF and the percentage of LDTF$^+$ cells in each MP cell cluster defined in Fig. 6A. Data are re-analyzed from previously deposited datasets (GSE145999) as detailed in Materials and Methods section. (**B**) In Fig. 7D, mRNA expression of signature genes along with Th1 (top) and Treg (bottom) trajectories is displayed.

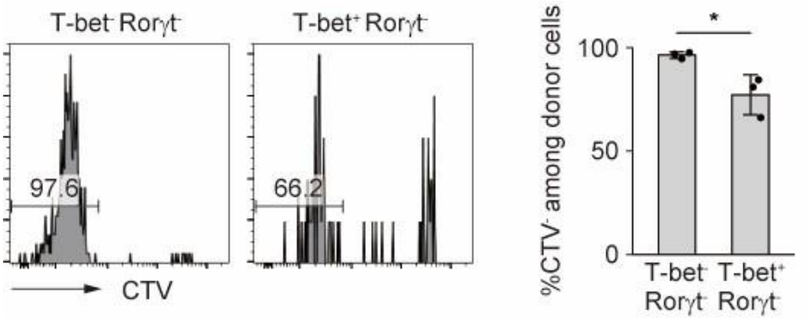

Fig. S7. T-bet$^-$ MP cells more rapidly proliferate in *Rag2$^{-/-}$* mice.

FACS-sorted, CTV-labeled T-bet$^-$ Rorγt$^-$ and T-bet$^+$ Rorγt$^-$ MP cells were separately transferred into *Rag2$^{-/-}$* mice and measured for their CTV dilution one week later. The representative histograms depict CTV dilution of donor cells in the spleen whereas the bar graph shows the frequency of CTV$^-$ cells among the total donor population (n=3). Bar graph: mean ± S.D, each symbol represents an individual mouse. * $p<0.05$.

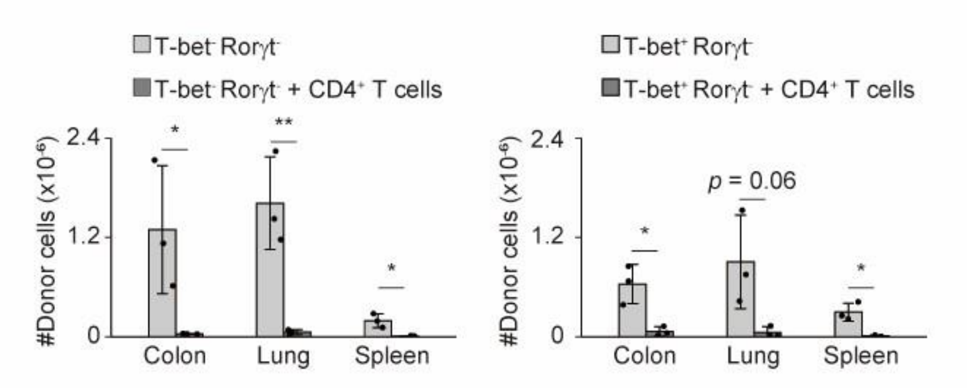

Fig. S8. Pre-existing CD4⁺ T lymphocytes inhibit expansion of MP cells in *Rag2*$^{-/-}$ mice.

Sorted T-bet$^-$ Rorγt$^-$ and T-bet$^+$ Rorγt$^-$ MP cells were separately transferred into *Rag2*$^{-/-}$ mice that had received total CD4$^+$ T lymphocytes 3 weeks in advance or been left intact. The bar graph shows the number of MP donor cells accumulating in the indicated organs 3 weeks after MP cell transfer (n=3). Pooled from two independent experiments. Bar graphs: mean ± S.D, each symbol represents an individual mouse. * $p<0.05$, ** $p<0.01$.

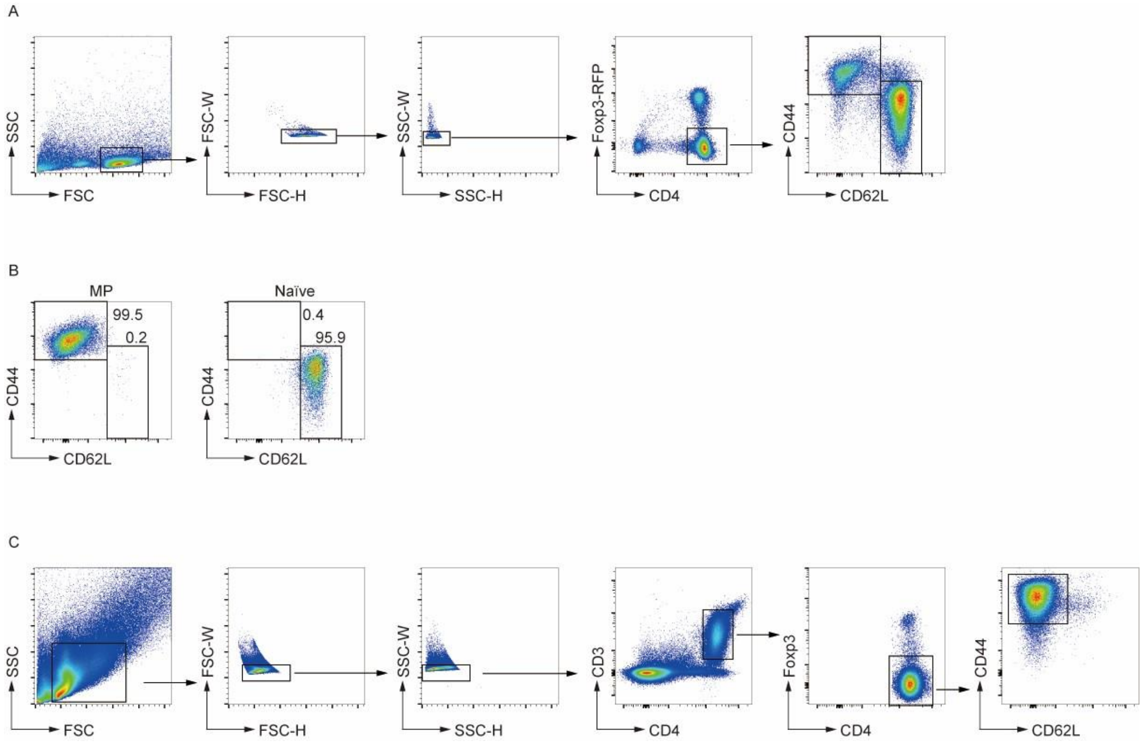

Fig. S9. Gating strategy.

(**A, B**) To sort for MP and naïve $CD4^+$ T lymphocytes, total singlet cells were gated for $CD4^+$ Foxp3-RFP^- cells. MP and naïve cells were then defined as $CD44^{hi}$ $CD62L^{lo}$ and $CD44^{lo}$ $CD62L^{hi}$ subpopulations, respectively. Post-sort purity was >95%. (**C**) To analyze donor cells in transfer experiments, total singlet cells were gated for $CD3^+$ $CD4^+$ $Foxp3^-$ $CD44^{hi}$ $CD62L^{lo}$ subpopulation.

**Th1**	**Th17**	**Treg**	**ISG^{hi}**
Ccr5	*Ccr6*	*Ctla4*	*Ifit1*
Cxcr3	*Il1r1*	*Foxp3*	*Ifit3*
Ifng	*Il1rap*	*Il2ra*	*Irf1*
Il12rb1	*Il17a*	*Il10*	*Irf4*
Il12rb2	*Il17f*	*Tnfrsf4*	*Isg15*
Il18r1	*Il22*		*Mx1*
Il18rap	*Il23r*		*Stat1*
Tbx21	*Rorc*		

Table S1. T cell gene signatures.

Data S1. Data values.
